# Speckle-free holography with partially coherent light sources and camera-in-the-loop calibration

**DOI:** 10.1126/sciadv.abg5040

**Published:** 2021-11-12

**Authors:** Yifan Peng, Suyeon Choi, Jonghyun Kim, Gordon Wetzstein

**Affiliations:** 1 Department of Electrical Engineering, Stanford University, 350 Jane Stanford Way, Stanford, CA 94305, USA.; 2 NVIDIA, 2788 San Tomas Expressway, Santa Clara, CA 95051, USA.

## Abstract

Computer-generated holography (CGH) holds transformative potential for a wide range of applications, including direct-view, virtual and augmented reality, and automotive display systems. While research on holographic displays has recently made impressive progress, image quality and eye safety of holographic displays are fundamentally limited by the speckle introduced by coherent light sources. Here, we develop an approach to CGH using partially coherent sources. For this purpose, we devise a wave propagation model for partially coherent light that is demonstrated in conjunction with a camera-in-the-loop calibration strategy. We evaluate this algorithm using light-emitting diodes (LEDs) and superluminescent LEDs (SLEDs) and demonstrate improved speckle characteristics of the resulting holograms compared with coherent lasers. SLEDs in particular are demonstrated to be promising light sources for holographic display applications, because of their potential to generate sharp and high-contrast two-dimensional (2D) and 3D images that are bright, eye safe, and almost free of speckle.

## Teaser

A holographic display combines artificial intelligence with partially coherent light sources to reduce speckle.

## INTRODUCTION

Holography is a technology with transformative potential in many display applications. For direct-view displays, holography enables glasses-free three-dimensional (3D) display modes. For near-eye displays used in virtual and augmented reality, holographic display modes have the potential to overcome long-standing challenges, such as optimizing perceived realism and visual comfort by solving the vergence-accommodation conflict (*1*, *2*). For heads-up displays, for example, in automotive applications, holographic displays provide unprecedented image brightness and dynamic range in addition to natural focus cues. Despite recent progress in optical systems and algorithms for computer-generated holography (CGH) (*3*), a fundamental challenge in making holography a practical alternative to conventional display technology is the speckle created by the coherent light sources used by virtually all holographic displays. Speckle is created by constructive and destructive interference of coherent light (*4*, *5*), and, in the aforementioned applications, it is not only perceived as severely degrading the image quality but it is also a potential safety hazard for the user (*6*).

Speckle reduction techniques often superimpose independent speckle patterns using either temporal or spatial multiplexing (*7*–*9*). These multiplexing methods include the use of mechanical vibration (*10*), fast-scanning micromirrors (*11*), deformable mirrors (*12*), and optically averaging different speckle patterns with varying phase delays (*13*). However, almost all multiplexing methods require either mechanically moving parts, complex optical systems, or both. Using partially coherent light sources, such as light-emitting diodes (LEDs), is attractive because it requires no hardware changes compared to using coherent sources (*14*–*17*). The spatial and temporal incoherence of LEDs directly reduces observed speckle using an effect that could be interpreted as “multiplexing” over multiple different wave propagation directions (i.e., spatial incoherence) or the spectrum (i.e., temporal incoherence). However, these same characteristics also degrade the observed image quality by introducing unwanted blur and loss of contrast. Recent CGH algorithms have attempted to precompensate this blur using optimization strategies (*18*, *19*) albeit with moderate success, partly because this is an ill-posed inverse problem. The overall image quality of LED-based holographic displays to date is very low and not comparable to that of recent coherent solutions. State-of-the-art CGH algorithms (*20*–*24*) lack appropriate mathematical models for the wave propagation from a partially coherent light source to a spatial light modulator (SLM) and to a target image.

**Fig. 1.**
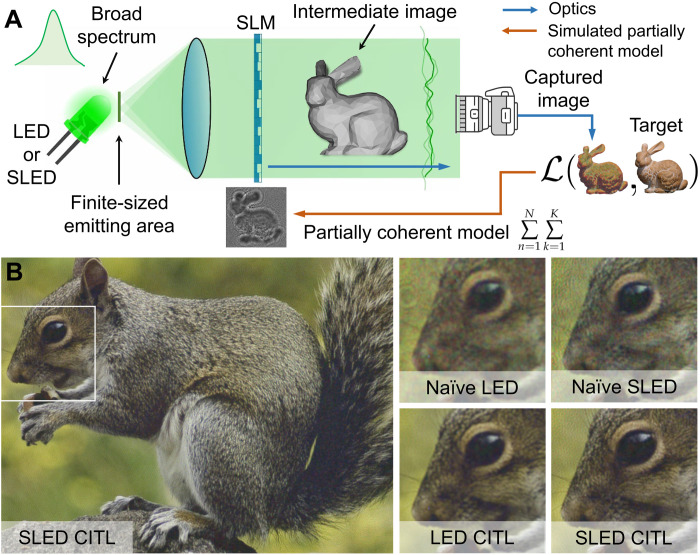
Overview of holography with partially coherent light sources using CITL calibration. (**A**) SLM phase patterns are iteratively shown, and the corresponding images were recorded by a camera. The error with reference to a target image is backpropagated into the phase pattern using the gradients of our partially coherent wave propagation model that considers a source of finite size and broad emission spectrum. (**B**) Experimentally captured 2D holographic images. Compared with holograms computed by a naïve wave propagation model, the CITL procedure optimizes image quality. Images Credits: Greg Turk and Marc Levoy, Stanford University, and Eirikur Agustsson and Radu Timofte, ETH Zurich.

Here, we develop a partially coherent wave propagation model that we use in conjunction with a modified version of a recently proposed camera-in-the-loop (CITL) calibration technique (see Fig. 1) (*23*). This approach allows us to achieve unprecedented experimental quality for 2D and multiplane 3D holographic images created by temporally and spatially incoherent LED light sources. Moreover, we experimentally validate that spatially coherent but temporally incoherent superluminescent LEDs (SLEDs) can further improve the image sharpness over LEDs without creating the speckle observed with coherent lasers.

## RESULTS

### Experimental setup

**Fig. 2.**
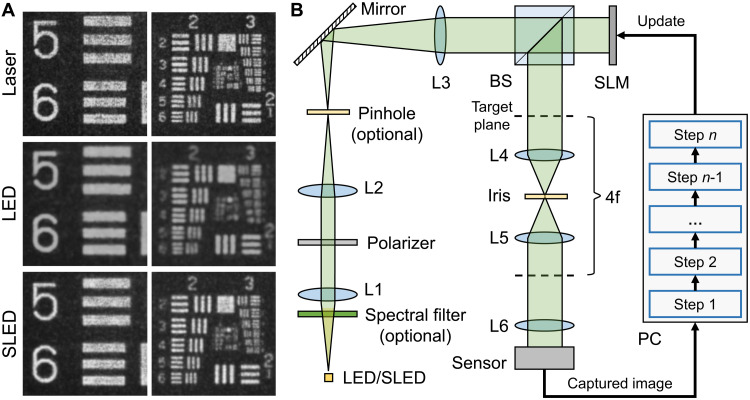
Resolution chart results shown on the holographic display setup. We present (**A**) close-up photographs of a resolution chart displayed with various light sources and (**B**) illustration of the holographic display setup. These holograms are experimentally captured for only the green color channel and visualized in grayscale. Note that the pinhole and spectral filter on the illumination path are only needed when using an LED as the light source.

For our experiments, we develop a holographic display prototype that is illustrated in Fig. 2B. We use a phase-only SLM (HOLOEYE LETO) with a 6.4-μm pixel pitch. The collimating lens (L3) is an achromatic doublet with a focal length of 200 mm. The eyepiece (L6) is a Nikon AF-S 50-mm f/1.4D lens. Two similar Nikon lenses (L4 and L5) are configured as a 4f system for filtering higher-diffraction orders with a 4-mm iris. Other components include a polarizer (Thorlabs, WP25M-VIS) and a beam splitter (BS; Thorlabs, BS016). All results are captured with a FLIR Grasshopper3 2.3 MP color USB3 vision sensor through a Nikon AF-S NIKKOR 35-mm f/1.8 lens. The CITL optimization is run on each color channel separately, and full-color results are combined in postprocessing.

For our baseline comparisons with a coherent laser source, we use a FISBA RGBeam fiber-coupled module with three optically aligned laser diodes and a maximum output power of 50 mW. The partially coherent light sources we evaluate include a white mounted LED (Thorlabs, MNWHL4f) with a maximum output power of 880 mW. The LED is coupled into a multimode fiber with a diameter of 200 μm, a pinhole with a diameter of 75 μm, and a single laser line filter with a 25.4 mm diameter. For the results using the LED source, all three color channels are captured sequentially using laser line filters with central wavelengths at 633, 532, and 460 nm, respectively, which are digitally combined in postprocessing. The full width at half maximum (FWHM) of these filters is 10 nm. In addition, we evaluated an SLED module (EXALOS RGB-SLED engines) that contains three aligned diodes and that is coupled into a single-mode fiber with a maximum output power of 5 mW. The central wavelengths are at 635, 510, and 450 nm, respectively. To account for the vastly different brightness of our light sources, we had to adjust the exposure time of our camera and increase it by a factor of 60× and 4× for the LED and SLED, respectively, relative to the laser source.

We implement all of our source code in PyTorch and sample 12 tuples over the spectrum and area of the LED or, alternatively, over just the spectrum of the SLED. Additional hardware and software details are discussed in Methods.

### Image formation model

At the core of the proposed approach is a partially coherent wave propagation model. For this purpose, let us first consider the wave field created by a coherent light source on the SLM plane *u*_src_(*x*, *y*, λ). Modulating this field by the SLM phase and propagating it in free space by distance *z* can be performed using the angular spectrum method (*25*, *26*) as

$$\begin{array}{c} {\hat{g}}_{c}(\phi ,u_{\text{src}},\lambda )=\iint F(e^{i\phi (x,y,\lambda )}u_{\text{src}})H(k_{x},k_{y},\lambda )e^{i2\pi (k_{x}x+k_{y}y)}{\mathrm{dk}}_{x}{\mathrm{dk}}_{y},\\ H(k_{x},k_{y},\lambda )=\{\begin{array}{cc} e^{i\frac{2\pi }{\lambda }\sqrt{1-{\left(\lambda k_{x}\right)}^{2}-{\left(\lambda k_{y}\right)}^{2}}z}, & \text{if}\;\sqrt{k_{x}^{2}+k_{y}^{2}}<\frac{1}{\lambda }\\ 0 & \text{otherwise} \end{array} \end{array}$$ (1)

where λ is the wavelength, *k_x_* and *k_y_* are spatial frequencies, ϕ is the wavelength-dependent per-pixel phase delay of the phase-only SLM, ℋ is the transfer function, ℱ(·) denotes the Fourier transform, and ${\hat{g}}_{c}$ is the coherent free space wave propagation operator ignoring any optical aberrations, SLM phase nonlinearities, or other electro-optical imperfections.

A partially coherent light source may have both a finite-sized area over which it emits light (i.e., it is spatially incoherent) and also a reasonably broad emission spectrum *q*(λ) (i.e., it is temporally incoherent). We thus model the continuous, and the approximated discrete, propagation of a partially coherent wavefield as

$$\begin{array}{cc} {|\;\hat{g}(\phi )|}^{2} & =∭q(\lambda )w(\omega ){|{\hat{g}}_{c}(\phi ,e^{i(\omega_{x}x+\omega_{y}y)},\lambda )|}^{2}\;d\lambda d\omega_{x}d\omega_{y}\\ & \approx \sum_{n=1}^{N}\sum_{k=1}^{K}q(\lambda^{\left(n\right)})w(\omega^{\left(k\right)}){|{\hat{g}}_{c}(\phi ,e^{i(\omega_{x}^{\left(k\right)}x+\omega_{y}^{\left(k\right)}y)},\lambda^{\left(n\right)})|}^{2} \end{array}$$ (2)

The discrete approximation samples wavelengths λ^(*n*)^ and angles **ω**^(*k*)^ over indices *n* and *k*. Here, *e*^*i*(ω*_x_x* + ω*_y_y*)^ models a tilted plane wave propagating into direction **ω** = (ω*_x_*, ω*_y_*), and *w* is the relative intensity of the source field along direction **ω**.

Using this model, we seek to solve a phase retrieval problem that takes partial coherence into account to minimize the loss ℒ between the amplitude of a holographic image and that of a target *a*_target_

$$\underset{\phi }{\text{minimize}}\;L(s\cdot |\hat{g}(\phi )|,a_{\text{target}})$$ (3)

where *s* is a scale factor that accounts for possible differences in the magnitudes of the simulated and target amplitudes. To solve Eq. 3, we use a stochastic gradient descent (SGD) solver. Unlike previous work that also used SGD for CGH applications (*23*, *27*), our problem is more challenging because of the integration of the wavelength spectrum and the range of angles. These integrals make our problem formulation more akin to a deconvolution problem embedded in a phase retrieval problem rather than a coherent phase retrieval problem alone.

The discrete model requires a sufficient number of samples and is memory demanding. In practice, for each iteration, we dynamically sample *M* tuples {**ω**^(*m*)^, λ^(*m*)^} uniformly over the finite size of the source (or physical pinhole) and its spectral emission profile (see the Supplementary Materials for details). This discrete model approximates the continuous wave propagation better with an increasing number of samples *M*, as in Monte Carlo integration. In practice, however, we found that sampling only a few tuples per iteration results in good convergence.

### Inversion procedure

Following Peng *et al.* (*23*), we use a CITL optimization strategy to mitigate the mismatch between the simulated wave propagation model $\hat{g}$ and the physical light transport of the display *g*, which includes optical aberrations, SLM phase nonlinearities, and other imperfections that degrade the image quality. Using a camera in the loop, this approach captures the holographic image for some displayed SLM phase pattern and backpropagates the error with reference to a target image into the phase pattern using the gradients of the ideal propagation model (see Fig. 1). Thus, the physical wave propagator *g* is used for the forward pass, and the gradients of the simulated model $\hat{g}$ are used for the backward pass. Specifically, the CITL method starts with some initial guess ϕ^(0)^ and then iterates as

$$\phi^{\left(k\right)}\leftarrow \phi^{\left(k-1\right)}-\alpha \;{\left(\frac{\partial L}{\partial |g|}\cdot \frac{\partial |\hat{g}|}{\partial \phi }\right)}^{T}\;L\;(s\cdot |g(\phi^{\left(k-1\right)})|,a_{\text{target}})$$ (4)

This gradient descent–type iteration scheme uses a user-defined step length of α. Note that our approach to CITL hologram optimization is unique in using a partially coherent wave propagation model that results in very different gradients from previously explored coherent models (*23*).

Figure 2A shows a comparison of a United States Air Force-1951 resolution chart, displayed by the same holographic display with all three light sources. Whereas the laser and SLED results are noticeably sharper than the LED result, the speckle exhibited by the laser is the strongest and that of the LED is the weakest. The SLED provides the best trade-off in being capable of displaying sharp images with a significantly lower amount of speckle than the laser.

We evaluate the effectiveness of our partially coherent model with an experimental ablation study in Table 1 (also see the Supplementary Materials). Here, the laser source is the baseline, providing a peak signal-to-noise ratio (PSNR) of 21.3 dB. The laser uses a coherent model, which is equivalent to our partially coherent model with a temporal bandwidth corresponding to a Dirac delta function δ_λ_ and an infinitesimal small source area δ_A_. Using the same coherent model to optimize holograms for LED or SLED sources achieves suboptimal results. By accounting for both spatial and temporal incoherence of the LED, the resulting PSNR can be maximized, even above that achieved by the laser. The SLED is spatially coherent, so we only need to model temporal incoherence, which, in this example, achieves the best results overall. Note that all results in Table 1 are experimentally captured using the CITL technique. This implies that the choice of model only influences the gradients that are used for backpropagating the error of the captured image with reference to the target image into the next phase pattern (cf. Eq. 4). As demonstrated, using gradients that best approximate those of the inaccessible physical wave propagation, by choosing an appropriate proxy model, is crucial.

**Table 1.** Ablation study evaluating the effectiveness of our partially coherent model. For the LED and SLED sources, we test our model with an increasing spectral bandwidth ranging from a Dirac delta δ_λ_ to 15 nm. The LED is further tested, assuming an infinitesimal small source with area δ_A_ and a finite size corresponding to our optical pinhole with a diameter of 75 μm. The PSNR (decibels) values significantly increase for the partially coherent sources when accounting for temporal and spatial incoherence. The SLED achieves the best result overall.

	**LED**		**SLED**	**Laser**
**Bandwidth ↓,** **Area →**	**δ_A_**	**75 μm**	**δ_A_**	**δ_A_**
δ_λ_	19.4	21.0	20.7	**21.3**
5 nm	20.3	21.3	21.4	
15 nm	21.0	**21.6**	**22.4**	

**Fig. 3.**
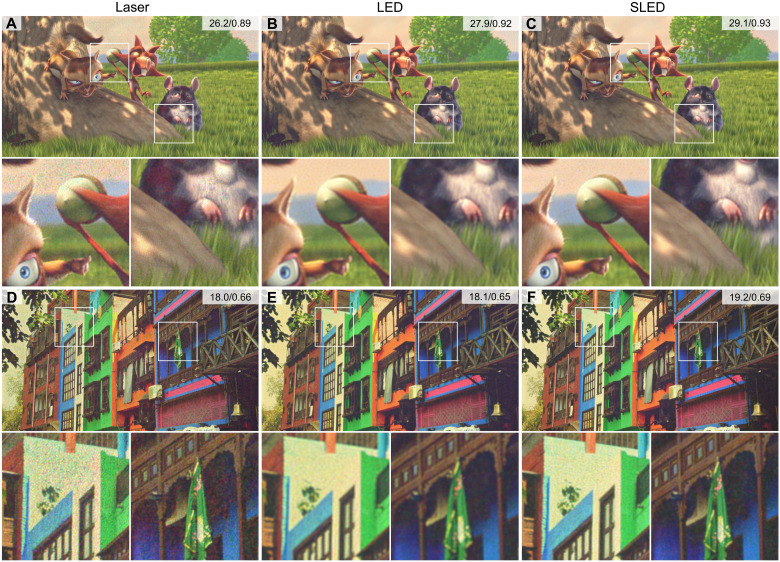
Experimentally captured 2D holographic images with different light sources. We use a coherent laser (**A** and **D**) and partially coherent LED (**B** and **E**) and SLED (**C** and **F**) light sources. All results are computed with the CITL optimization described in the main text. Metrics represent PSNR (in decibels) and structural similarity, respectively. In both examples, the laser and SLED sources achieve sharper image features than the LED. However, the laser creates speckle that cannot be removed in the software alone. The combination of SLED source and CITL algorithm successfully removes the remaining speckle while achieving sharp image detail. Images Credits: Big Buck Bunny, Blender Institute, and Eirikur Agustsson and Radu Timofte, ETH Zurich.

Figure 3 shows experimentally captured 2D holographic images with all three light sources. All of these results are captured with the discussed CITL calibration, and the LED and SLED results use the appropriate partially coherent model. The holograms captured with the coherent laser (Fig. 3, A and D) not only show sharp, high-contrast results but also significant speckle, which cannot be corrected by the CITL calibration. Both LED (Fig. 3, B and E) and SLED (Fig. 3, C and F) achieve almost speckle-free results, especially in uniformly colored image areas, such as the sky. However, the LED also introduces blur, which is not produced by the SLED. Note that the exposure time of our camera images is adjusted, as previously discussed, and that the LED is actually significantly dimmer than both of the other light sources. Again, the SLED yields the highest image quality with good sharpness and minimum speckle at a reasonable brightness.

**Fig. 4.**
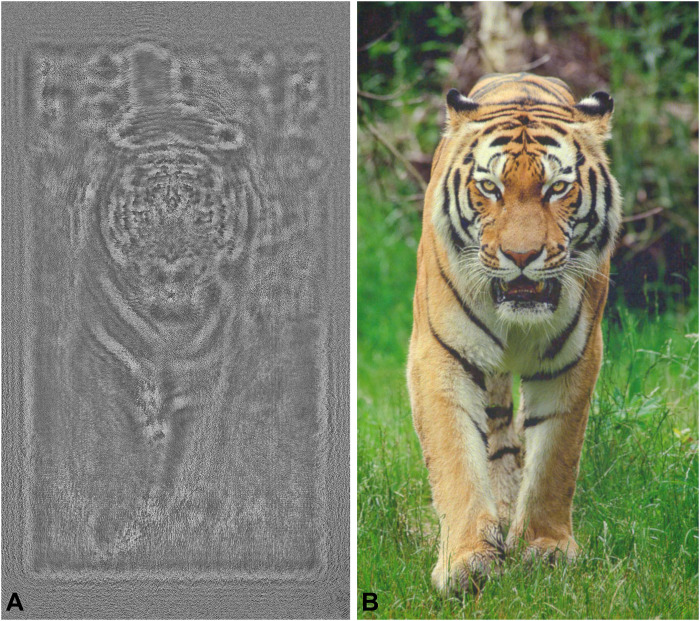
Experimentally captured 2D holographic image and corresponding phase pattern. We present the holographic image (**B**) obtained using SLED along with the SLM phase pattern (**A**) that was automatically optimized using the proposed CITL strategy. This result shows sharp image details without noticeable speckle artifacts. Image Credit: Eirikur Agustsson and Radu Timofte, ETH Zurich.

Figure 4 shows an additional result captured with the SLED source along with the corresponding SLM phase pattern our CITL calibration technique estimated. Image details are sharp, colors are crisp, and no speckle is observed. This captured holographic image is perhaps one of the highest-quality results demonstrated to date.

**Fig. 5.**
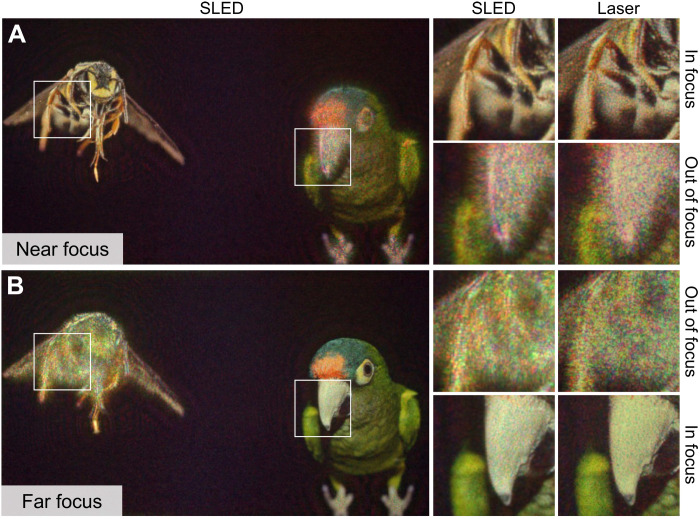
Experimental 3D results of SLED- and laser-based holography. The insect and the bird are located at a (**A**) near (0.5 m) and a (**B**) far plane (optical infinity). PSNR values for SLED and laser are 21.94 and 20.95 dB, respectively. Images Credits: Licensed under Creative Commons.

Last, we explore 3D holography with a two-plane setup in Fig. 5. In this example, the target objects are located at a near distance of 0.5 m and a far distance of optical infinity from the user. We use two differently focused cameras of the same model to capture both focal planes simultaneously and backpropagate the error of both planes to the same SLM phase pattern using our CITL procedure. As in the other examples, the SLED is capable of achieving in-focus results that are as sharp as the laser while reducing speckle. The out-of-focus areas are not constrained by our algorithm and are not noticeably different.

## DISCUSSION

In summary, we develop a partially coherent wave propagation model that is well suited for the CITL calibration procedure that was recently proposed for coherent light sources (*23*). We evaluate this new approach to CGH with an incoherent LED and a partially coherent SLED source. SLEDs are spatially coherent but temporally incoherent; similar to lasers, SLEDs emit collimated light with very low beam divergence, but, similar to LEDs, they emit light over a broad spectrum of wavelengths. Both of these types of light sources can noticeably reduce speckle compared to lasers, but the LED also creates slightly blurred images and is significantly dimmer than both laser and SLED. Overall, the SLED source combined with our new CGH algorithm provides the best trade-off between image sharpness and contrast, brightness, and speckle.

Our current prototype allows us to demonstrate the transformative potential of partially coherent holography for near-eye displays that magnify the small image of an SLM using an eyepiece. A miniaturized version of this setup would be directly applicable to virtual reality display applications. In future work, we would like to demonstrate this system with optical combiners used in optical see-through augmented reality and automotive heads-up display applications. To demonstrate the full potential of this technology, glasses-free 3D display would require larger-scale display panels with ultrasmall pixel pitches to enable the large space-bandwidth products required for those applications. While this is not possible with commercially available hardware, metasurface-based technology is making quick progress toward large-scale, high-resolution displays (*28*).

Another limitation of our approach is the requirement of a camera in the loop of the hologram optimization procedure. Our experiments demonstrate that this works well for optimizing holographic 2D images and multiplane 3D images with nonoverlapping regions at different depths. The ability of displaying holographic 3D images with continuous depth, however, would require a wave propagation model to be trained and used for the hologram optimization. While such models have been proposed for 2D holographic images (*23*), developing camera-calibrated 3D wave propagation models for partially coherent sources is an interesting avenue of future work. An additional benefit of such models would be the ability to remove the camera from the display system for computing holograms once the model is trained.

Our work develops modern, artificial intelligence–inspired algorithms for CGH to demonstrate the high-quality and speckle-free holographic 2D and 3D images using partially coherent light sources. We believe that our approach bridges the long-standing gap between computer-generated holographic display theory and practice and makes holographic displays a viable technology, particularly for emerging virtual and augmented reality applications.

## METHODS

### Details of experimental setup

Our holographic near-eye display setup uses two different types of partially coherent illumination sources, LEDs and SLEDs, in addition to conventional coherent lasers. The LED light engine comprises a white mounted LED (Thorlabs, MNWHL4f) with a maximum output power of 880 mW, a multimode fiber (Thorlabs, M72L01) with a diameter of 200 μm and an numerical aperture of 0.39, a pinhole with a diameter of 75 μm, and one of three laser line filters each with a 25.4 mm diameter for each of the color channels with central wavelengths at 633, 532, and 460 nm, respectively. The FWHM of the filters is 10 nm. The SLED module (EXALOS RGB-SLED engines) contains three aligned diodes and is coupled with a single-mode fiber with a maximum output power of 5 mW. The central wavelengths are at 635, 510, and 450 nm, respectively. The baseline laser for comparison experiments is a FISBA RGBeam fiber-coupled module with three optically aligned laser diodes with a maximum output power of 50 mW. In our implementation, color images are captured as separate exposures for each channel and then combined in postprocessing. Experimental characterizations of the coherence properties of both LEDs and SLEDs are shown in the Supplementary Materials.

The SLM is a Holoeye Leto phase-only liquid crystal on silicon with a resolution of 1920 by 1080 and a pixel pitch of 6.4 μm. This device provides a bit depth of 8 bits and a diffraction efficiency of over 80%. The eyepiece is a Nikon AF-S 50-mm f/1.4D lens (L6). Other components include a polarizer (Thorlabs, WP25M-VIS) and a BS (Thorlabs, BS016).

We further use a 4f system consisting of two Nikon 50-mm f/1.4D lenses (L4 and L5) and an iris with a diameter of 4 mm to optically filter our higher diffraction orders. Note that the mechanism does not filter out the undiffracted light (i.e., the direct current or DC component). All images are captured with a FLIR Grasshopper3 2.3 MP color vision sensor through a Nikon AF-S NIKKOR 35-mm f/1.8G lens. Captured images are processed on a PC to update the patterns displayed on the SLM.

### Software implementation

All CGH algorithms are implemented in PyTorch (*29*). Pseudo-code for SGD and CITL algorithms with the stochastic sampling are outlined in the Supplementary Materials. The homography used in the experiments follows the same procedure in the recent work (*23*). As a specific instance, with the SLED-based implementation on the graphics processing unit Nvidia RTX 2080Ti, the optimization process outlined in algorithms S1 and S2 takes about 100 and 480 s for 500 iterations, respectively. For all algorithm implementation in this work, we set the learning rate α to 0.006 for all phase variables and 0.001 for the scalar *s*, and we use the 𝓁_2_ loss function.

## Supplementary Materials

### This PDF file includes:

Derivation of Partially Coherent Hologram Synthesis Implementation Details of the Display Prototype Additional Results Figs. S1 to S20 References
