# Supplementary material for: Speckle-free holography with partially coherent light sources and camera-in-the-loop calibration
Source: Sci Adv. 2021 Nov 12;7(46):eabg5040. doi: 10.1126/sciadv.abg5040 (PMC8589315; doi:10.1126/sciadv.abg5040)
Supplement: Supplementary file 1 — Derivation of Partially Coherent Hologram Synthesis Implementation Details of the Display Prototype Additional Results Figs. S1 to S20 References [file sciadv.abg5040_sm.pdf]

Science Advances
AAAS

Supplementary Materials for

Speckle-free holography with partially coherent light sources and camera-in-the-loop calibration

Yifan Peng, Suyeon Choi, Jonghyun Kim, Gordon Wetzstein*

*Corresponding author. Email: gordon.wetzstein@stanford.edu

Published 12 November 2021, *Sci. Adv.* **7**, eabg5040 (2021)
DOI: 10.1126/sciadv.abg5040

This PDF file includes:

Derivation of Partially Coherent Hologram Synthesis
Implementation Details of the Display Prototype
Additional Results
Figs. S1 to S20
References

This supplementary document includes complementary derivations related to propagation and optimization models of a partially coherent holographic system, implementation details of both display hardware and computer-generated hologram algorithms, and additional experimental results.

Here we list the abbreviations and notations used across this document.

- **SLM:** a spatial light modulator
- **CGH:** computer-generated holography
- **LED:** light emitting diode
- **SLED**: super-luminescent light emitting diode
- **ASM:** the angular spectrum method [9]
- **CITL:** camera-in-the-loop optimization
- **SGD:** stochastic gradient descent–based phase retrieval
- **laser CITL:** an optical implementation that uses a laser and SGD-based CITL optimization
- **LED CITL:** an optical implementation that uses an LED and SGD-based CITL optimization
- **SLED CITL:** an optical implementation that uses an SLED and SGD-based CITL optimization
- **Coherent Model:** propagation model that considers only one single wavelength emitting from one spatial point
- **Partially Coherent Model:** propagation model that considers multiple wavelengths emitting from multiple spatial points

1. DERIVATION OF PARTIALLY COHERENT HOLOGRAM SYNTHESIS

Throughout this work, we consider an electronic holographic display system that uses a single phase-only SLM. In this system, either a coherent laser or a partially coherent source, such as an LED or SLED, is used. The coherent or partially coherent source field is incident on the SLM, where the phase is delayed in a spatially varying manner. The field continues to propagate in free space by some distance to a target plane, where a user or a detector observe the intensity. Thus, we consider a free space propagation–type holographic display system as opposed to a Fourier–type system.

A. Coherent Image Formation and Inversion

To model the free-space propagation between the source, i.e., the SLM, and the target planes, the ASM [9, 26] is applied. For a coherent source $u_{\text{src}}\left(x, y, \lambda\right)$, this model propagates the field by a fixed distance z as

$$\widehat{g}_c\left(\phi, u_{\text{src}}, \lambda\right) = \iint \mathcal{F}\left(u_{\text{src}}\left(x, y, \lambda\right) e^{i\phi(x,y,\lambda)}\right) \mathcal{H}\left(k_x, k_y, \lambda\right) e^{i2\pi(k_x x + k_y y)} dk_x dk_y\,,$$
$$\mathcal{H}\left(k_x, k_y, \lambda\right) = \begin{cases} e^{i\frac{2\pi}{\lambda}\sqrt{1-(\lambda k_x)^2-\left(\lambda k_y\right)^2}z}, & \text{if } \sqrt{k_x^2+k_y^2} < \frac{1}{\lambda} \\ 0 & \text{otherwise} \end{cases}, \tag{S1}$$

where λ is the wavelength, k_x and k_y are spatial frequencies, $\mathcal{H}$ is the transfer function, and $\mathcal{F}(\cdot)$ denotes the Fourier transform.

In this coherent case, a phase retrieval algorithm would then aim at estimating the SLM phase pattern ϕ that minimizes the error with respect to some target amplitude a_{target} by solving the optimization problem

$$\underset{\phi}{\text{minimize}}\ \mathcal{L}\left(s \cdot \left|\widehat{g}_c\left(\phi\right)\right|, a_{\text{target}}\right), \tag{S2}$$

where $\mathcal{L}\left(\cdot\right)$ is a loss function, such as the ℓ_2 norm. Numerous approaches to solving phase retrieval problems of this form have been proposed in the literature [30]. However, even the most sophisticated algorithms cannot prevent speckle in the physical display system, because this is caused by the coherent property of the light source.

B. Partially Coherent Image Formation and Inversion

A partially coherent source, e.g., an LED or SLED, may have both a reasonably broad emission spectrum $q\left(\lambda\right)$ and also a finite-sized area over which it emits light with some spatial intensity profile. Thus, it can exhibit partial coherence in both the spatial and/or temporal domain. We assume that the light source is reasonably well collimated, such that the spatial emission profile directly maps to the angular profile of source field $w\left(\boldsymbol{\omega}\right)$, where $\boldsymbol{\omega} = \left(\omega_x, \omega_y\right)$ is a vector indicating a two-dimensional direction. The specific mapping from area to angle depends on the focal length of the lens that collimates the source (see Eq. S8). Taking this into account, we derive the image intensity $\hat{I}$ using a partially coherent image formation model $\widehat{g}$ as

$$\hat{I}\left(\phi\right) = \left|\widehat{g}\left(\phi\right)\right|^2 = \iiint q\left(\lambda\right) w\left(\boldsymbol{\omega}\right) \left|\widehat{g}_c\left(\phi, e^{i\left(\omega_x x + \omega_y y\right)}, \lambda\right)\right|^2 d\lambda d\omega_x d\omega_y, \tag{S3}$$

where $e^{i\left(\omega_x x + \omega_y y\right)}$ models a tilted plane wave propagating into direction $\boldsymbol{\omega}$, and w is the relative intensity of the source field along direction $\boldsymbol{\omega}$.

In practice, we deal with discrete signals, including the SLM phase ϕ, sampled wavelengths $\left\{\lambda^{(n)}\right\}_{n=1}^{N}$ and angles $\left\{\boldsymbol{\omega}^{(k)}\right\}_{k=1}^{K}$, so Eq. S3 can be represented as the weighted sum over spectral and spatial domains

$$\left|\widehat{g}\left(\phi\right)\right|^2 \approx \sum_{n=1}^{N} \sum_{k=1}^{K} q\left(\lambda^{(n)}\right) w\left(\boldsymbol{\omega}^{(k)}\right) \left|\widehat{g}_c\left(\phi, e^{i\left(\omega_x^{(k)} x + \omega_y^{(k)} y\right)}, \lambda^{(n)}\right)\right|^2. \tag{S4}$$

A phase retrieval algorithm that takes partial coherence into account can then be formulated as

$$\underset{\phi}{\text{minimize}}\ \mathcal{L}\left(s \cdot \left|\widehat{g}\left(\phi\right)\right|, a_{\text{target}}\right). \tag{S5}$$

Problem S5 can be efficiently solved with many existing approaches. In this work, we employ the SGD algorithm because it has recently been demonstrated as an intuitive and robust solver for computer-generated holography problems [23]. What makes this problem challenging is the integration over the wavelength spectrum and also over the incident angles—the very properties that provide the benefit of speckle reduction. These integrals make this partially coherent formulation more akin to a deconvolution problem embedded in a phase retrieval problem than the coherent phase retrieval problem alone. Next, we describe how to practically characterize the propagation in discrete spectral and spatial domain, respectively.

Derivation for temporal incoherence For a broadband incident beam emitting from a point source, and ignoring the source field u_{src}, the forward wave propagation model incorporates a sum of propagated intensity over multiple sampling wavelengths

$$\left|\widehat{g}_t(\phi)\right|^2 = \sum_{n=1}^{N} q(\lambda_n) \left|\mathcal{F}^{-1}\left(\mathcal{F}\left(e^{i\phi}\right) \cdot \mathcal{H}_{\lambda_n}\right)\right|^2, \tag{S6}$$

where $\mathcal{H}_{\lambda_n}$ is the transfer function for wavelength λ_n.

Derivation for spatial incoherence Here, we consider a single wavelength and multiple mutually incoherent sources that are laterally shifted at the source plane. The wave emitting from an ideal point source at the center, i.d., $\delta(\mathbf{r_0})$, is first collimated by a collimating lens and hits the SLM as a plane wave

$$\underbrace{a(\mathbf{r_0})\delta(\mathbf{r_0}-0)}_{\text{source plane}} \rightarrow \underbrace{u_{\text{src}}(\mathbf{r}|\mathbf{r_s}=0) = c \cdot a\,(0)}_{\text{SLM plane}}, \tag{S7}$$

where a indicates the spatial amplitude profile of the finite source, $\mathbf{r}$ and $\mathbf{r_0}$ are the coordinates at the SLM plane and source plane, respectively. $\mathbf{r_s}$ indicates a lateral shift of the point light source, which is 0 here, and c is a constant.

If the point light source is shifted for $\mathbf{r_s} \neq 0$, the wave incident on the SLM becomes a tilted planar wave [31], indicated as

$$\underbrace{a(\mathbf{r_0})\delta(\mathbf{r_0}-\mathbf{r_s})}_{\text{source plane}} \rightarrow \underbrace{u_{\text{src}}(\mathbf{r}|\mathbf{r_s}) = c \cdot a\,(\mathbf{r_s})\, e^{2\pi i \frac{\mathbf{r}\cdot\mathbf{r_s}}{\lambda f_c}}}_{\text{SLM plane}}, \tag{S8}$$

where f_c is the focal length of the collimating lens. Again, this implies that the lateral shift of light source leads to the tilted plane wave with direction $\boldsymbol{\omega} = 2\pi\frac{\mathbf{r_s}}{\lambda f_c}$ and the angular profile of source intensity w maps to the spatial amplitude profile at the pinhole as $w\,(\boldsymbol{\omega}) = (c \cdot a\,(\mathbf{r_s}))^2$.

A finite-sized emitting area can be represented as a set of many incoherent point sources. Thus, contributions from angles $\{\omega_k\}_{k=1}^{K}$ are added up in intensity to derive the spatial incoherence as

$$|\widehat{g}_s(\phi)|^2 = \sum_{k=1}^{K} w\,(\boldsymbol{\omega}_k) \left| \mathcal{F}^{-1}\left(\mathcal{F}\left(e^{i\omega_k\cdot\mathbf{r}}e^{i\phi}\right)\cdot\mathcal{H}\right)\right|^2, \tag{S9}$$

The image intensity emitting from a shifted point light source results in the laterally shifted image intensity from the centered point light source and it can be calculated using the convolution with a spatially invariant kernel. Relevant details can be found in the literature [14, 18, 19].

Stochastic sampling over spectral and spatial domain The discrete partially coherent propagation model described in Eq. S4 requires a sufficient number of samples and it is memory demanding since it calculates a wave propagation NK times. In practice, for each iteration, we dynamically sample M tuples $\left(\boldsymbol{\omega}^{(m)}, \lambda^{(m)}\right)$ uniformly over the finite size of the source (or physical pinhole) and the characterized spectra, which is shown in Figure S5. Then, we weigh the emission spectrum $q\left(\lambda^{(m)}\right)$ and angular profile of source intensity $w(\boldsymbol{\omega}^{(m)})$ on the propagated intensity as in Eq. S4 and sum them up for all M pairs. One can pre-compute N (>M) tuples of $\left(\boldsymbol{\omega}^{(m)}, \lambda^{(m)}\right)$ and set them as a pool, and randomly pick from the pool every iteration, which gives significant speedup. We show the convergence of the optimization with different light sources and propagation models in Figure S1.

C. Camera-in-the-loop Hologram Optimization

It is clear that the mismatch between the simulated wave propagation model $\widehat{g}$ (Eqs. S1, S5) and the physical wave propagation of the display g drastically influences the holographic image quality. Optical aberrations, phase nonlinearties of the SLM pixels, source intensity variation, diffraction efficiency of the SLM and many other factors typically degrade the image quality of experimental results of holographic display systems compared to comparable simulations.

To mitigate this discrepancy, Peng et al. [23] recently introduced a camera-in-the-loop hologram optimization strategy. Their approach builds on the insight that an SGD optimization strategy for problems like Eqs. S2 or S5 can use a camera to have the optimization work directly with g and parts of its gradients for many of the required calculations, rather than relying on the approximate model $\widehat{g}$. Specifically, a CITL-type gradient descent solver starts with some initial guess $\phi^{(0)}$, we use

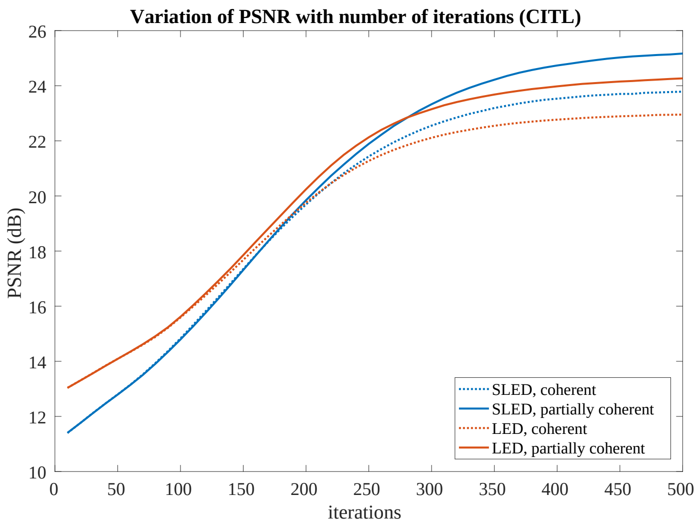

Fig. S1. Convergence graph of CITL optimization with different light sources and propagation models. This evaluation is run on the red channel. The PSNRs are averaged over 5 images at each iteration. Note that the coherent/partially coherent models only differ in their gradients since the CITL optimization uses captured images for forward propagation. Our partially coherent model leads to a performance improvement of around 1.5 dB when using SLED and LED.

random values, and then iterates as

$$\begin{aligned} \phi^{(k)} \leftarrow \phi^{(k-1)} &- \alpha \left(\frac{\partial \mathcal{L}}{\partial \phi}\right)^T \mathcal{L}\left(s \cdot \left|g\left(\phi^{(k-1)}\right)\right|, a_{\text{target}}\right) \\ \approx \phi^{(k-1)} &- \alpha \left(\frac{\partial \mathcal{L}}{\partial |g|} \cdot \frac{\partial |\widehat{g}|}{\partial \phi}\right)^T \mathcal{L}\left(s \cdot \left|g\left(\phi^{(k-1)}\right)\right|, a_{\text{target}}\right). \end{aligned} \tag{S10}$$

Note that both the forward image formation model $\left|g\left(\phi^{(k-1)}\right)\right|$ and the partial derivative $\frac{\partial \mathcal{L}}{\partial |g|}$ (formulated as a Jacobian matrix) are computed with the captured camera image using the physical image formation g. Thus, the only part where a model mismatch occurs here is in the term $\frac{\partial |\widehat{g}|}{\partial \phi}$, which uses the gradients of the proxy model from Eq. S5 for the backpropagation pass, because these are physically inaccessible by the camera. s is a scale factor that can contribute to bridging the gap between the captured and target amplitudes. We adopt this CITL optimization strategy for our partially coherent image formation and implement the forward model as well as the optimization routines in PyTorch, which makes it easy to have Pytorch's automatic differentiation engine calculate the partial derivatives of the proxy model. Refer to the next section for implementation details.

2. IMPLEMENTATION DETAILS OF DISPLAY PROTOTYPE

A. Hardware Implementation

As shown in Figure S2, we build a benchtop holographic near-eye display setup using *two* types of partially coherent illumination sources—LED and SLED.

For the first demonstration, we use a white mounted LED (Thorlabs MNWHL4f) with a maximum output power of 880 mW, a multi-mode fiber (Thorlabs M72L01) with a diameter of 200 μm and an NA of 0.39, a pinhole with a diameter of 75 μm, and one of three laser line filters with a 1 inch diameter and with their central wavelengths at 633 nm, 532 nm, and 460 nm, respectively. The FWHM of the filters is 10 nm. For another demonstration, we use a SLED module (EXALOS RGB-SLED engines) that contains three aligned diodes and is coupled with a single-mode fiber with a maximum output power of 5 mW. The central wavelengths are at 635 nm, 510 nm, and 450 nm, respectively.

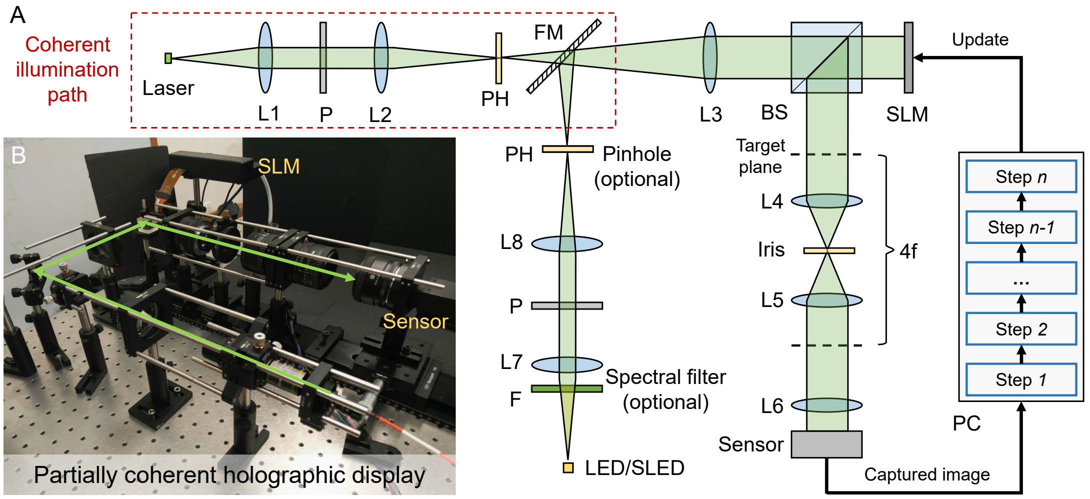

Fig. S2. Hardware implementation of our partially coherent holographic display. We show the schematic diagram (A) and setup photograph (B). Specifically, L: lens, F: color filter (optional), P: linear polarizer, PH: pinhole, FM: flip mirror, and BS: beam splitter. Note that we also build a coherent illumination path for comparison that uses a laser as the illumination source. The pinhole and filter on the illumination path are only needed when using an LED as the light source, but they are not used for the SLED. Photo Credit: Yifan Peng, Suyeon Choi, Jonghyun Kim, Gordon Wetzstein, Stanford University.

The collimating lens (L3 in Figure S2) is an achromatic doublet with a focal length of 200 mm. The baseline laser for comparison experiments is a FISBA RGBeam fiber-coupled module with three optically aligned laser diodes with a maximum output power of 50 mW. In our implementation, color images are captured as separate exposures for each channel and then combined in post-processing.

The SLM is a HOLOEYE LETO phase-only LCoS with a resolution of 1,920 $\times$ 1,080 and a pixel pitch of 6.4 μm. This device provides a bit depth of 8 bits and a diffraction efficiency of over 80%. The eyepiece is a Nikon AF-S 50 mm f/1.4D lens (L6). Other components include a polarizer (Thorlabs WP25M-VIS), and a beam splitter (BS, Thorlabs BS016). We further use a 4f system consisting of two Nikon 50 mm f/1.4D lenses (L4 and L5) and an iris with a diameter of 4 mm to optically filter out higher diffraction orders. Note that this mechanism does not filter out the undiffracted light (i.e., the direct current or DC component). All images are captured with a FLIR Grasshopper3 2.3 MP color vision sensor through a Nikon AF-S Nikkor 35 mm f/1.8G lens. Captured images are processed on a PC to update the patterns displayed on the SLM. As such, we have realized a partially coherent holographic display with camera-in-the-loop optimization.

We experimentally characterize the coherence property of both light sources. The spectral response curves of the customized partially coherent light sources are illustrated in Figure S3. In the optimization, we extract the weighting response q from these characterized plots.

B. Software Implementation

All CGH algorithms are implemented in PyTorch [29]. Pseudocode for SGD and CITL algorithms with the stochastic sampling are outlined in Algorithms S1 and S2, respectively. The homography used in the experiments follows the same procedure in the recent work [23]. As a specific instance, with the SLED-based implementation on the GPU Nvidia RTX 2080Ti, the optimizations process outlined in Algorithms S1 and S2 takes about 100 s and 480 s for 500 iterations, respectively. For all algorithm implementation in this work, we set the learning rate to 0.006 for all phase variables, 0.001 for the scalar, and we use the ℓ_2 loss function.

Algorithm S1. SGD Hologram Optimization with Partially Coherent Model

M : number of sampled pairs of wavelengths/source points for each iteration
$\texttt{model\_p}(\cdot|\,\{\lambda_m, \omega_m\}_{m=1}^{M})$: propagation through partially coherent model
$\texttt{model\_bp}(\cdot|\,\{\lambda_m, \omega_m\}_{m=1}^{M})$: backpropagation through partially coherent model
$\texttt{loss\_bp}(\cdot)$: backpropagation through loss function

foreach k **in** $1 \ldots K$ **do**
  randomly sample $\omega_1, \cdots, \omega_M, \lambda_1, \cdots, \lambda_M$
  $\widehat{g} \leftarrow \texttt{model\_p}(\phi|\,\{\lambda_m, \omega_m\}_{m=1}^{M})$
  $\{\phi, s\} \leftarrow \texttt{model\_bp}(\texttt{loss\_bp}(\mathcal{L}(s \cdot |\widehat{g}|, a_{\text{target}}))|\,\{\lambda_m, \omega_m\}_{m=1}^{M})$
return ϕ

Algorithm S2. CITL Hologram Optimization with Partially Coherent Model

M : number of sampled pairs of wavelengths/source points for each iteration
$\texttt{model\_p}(\cdot|\,\{\lambda_m, \omega_m\}_{m=1}^{M})$: propagation through partially coherent model
$\texttt{model\_bp}(\cdot|\,\{\lambda_m, \omega_m\}_{m=1}^{M})$: backpropagation through partially coherent model
$\texttt{loss\_bp}(\cdot)$: backpropagation through loss function
$\texttt{camera\_p}(\cdot)$: camera captured intensity (raw mode) + homography
$\texttt{replace}(s, t)$: replace values of s with t, retain gradients from s

foreach k **in** $1 \ldots K$ **do**
  randomly sample $\omega_1, \cdots, \omega_M, \lambda_1, \cdots, \lambda_M$
  $\widehat{g} \leftarrow \texttt{model\_p}(\phi|\,\{\lambda_m, \omega_m\}_{m=1}^{M})$
  $|g| \leftarrow \texttt{replace}(|\widehat{g}|, \sqrt{\texttt{camera\_p}(\phi)})$
  $\{\phi, s\} \leftarrow \texttt{model\_bp}(\texttt{loss\_bp}(\mathcal{L}(s \cdot |g|, a_{\text{target}}))|\,\{\lambda_m, \omega_m\}_{m=1}^{M})$
return ϕ

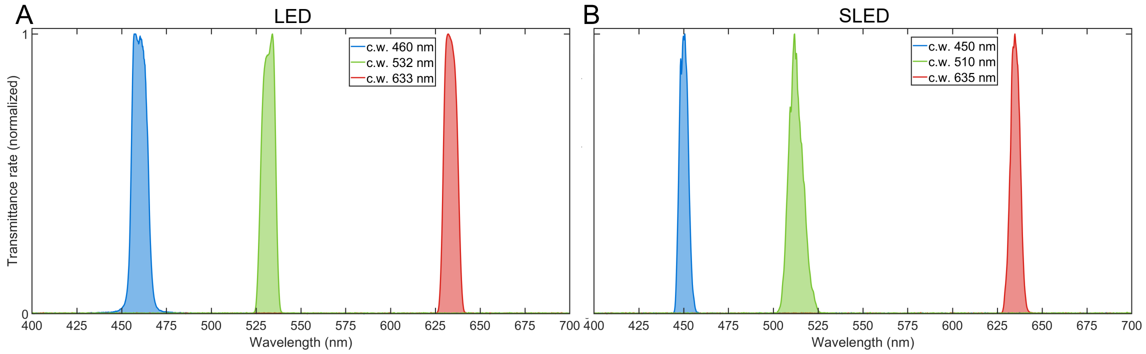

Fig. S3. Characterized spectra of three color channels for two partially coherent light sources. The characterized light sources include (A) LED and (B) SLED. The central wavelengths (c.w.) of three bands are indicated.

3. ADDITIONAL RESULTS

Ablation study on temporal and spatial incoherence modeling To fully explore the impact of the pinhole size on the spatial coherence and on the perceptual quality, we conduct an ablation study. In Figure S4, we present comparison results using the laser, SLED, LED + 100 μm pinhole, LED + 75 μm pinhole, and LED + 50 μm pinhole, respectively. We observe that decreasing the pinhole size leads to stronger spatial coherence that further contributes to a less ill-posed optimization problem. However, a smaller pinhole comes at the cost of sacrificing light efficiency. We experimentally observe that a 75 μm can provide an almost comparable display quality for an LED with reference to laser-based holography. Importantly, speckle artifacts are significantly mitigated. In these experiments an achromatic doublet with a focal length of 200 mm is used to collimate the light emitting from the pinhole.

Next, we present a more comprehensive study with different sampling schemes along the wavelength and angle diversity (see Figure S5), as the complementary to Table 1 in the main text. We observe that when only sampling one wavelength and one source point for the LED, the results exhibit blur and low contrast. Increasing the number of samples in either spectral or spatial domain leads to improved image contrast as well as higher spatial resolution. The full partially coherent model leads to the best reconstruction quality, i.e., the highest PSNR. However, comparing with the target image, partially coherent holography with the LED still exhibits an expected amount of resolution and contrast loss. In contrast, using an SLED leads to a much better compromise between speckle and resolution (see the bottom row of Figure S5).

Analyzing SLED Bandwidth and LED Pinhole Size Our results, especially the ablation study shown in the previous subsection, indicate that there may be an optimal spectral bandwidth of an SLED source and an optimal pinhole size of the LED. In this subsection, we aim at answering the following questions: (1) what is the optimal bandwidth of an SLED?; (2) what is the optimal pinhole size for an LED with an extremely narrow bandwidth?; (3) what is the optimal pinhole size for an LED with a moderate bandwidth?

To answer these questions, we perform three experiments in simulation. For all of these, we compare the results achieved by a conventional SGD solver and our CITL approach. Here, SGD uses our partially coherent forward model (Eq. S4) to simulate the forward pass from phase-only SLM pattern to intensity on the target plane and it also uses the gradients of this partially coherent model for the error backpropagation pass. With the SGD approach, we optimize the SLM phase pattern using this partially coherent model and then we simulate how this phase pattern would look on a physical display by introducing some amount of mismatch between the wave propagation model and the physical optics. Specifically, we simulate this mismatch by introducing zero-mean Gaussian noise with a standard deviation of $\sigma = 0.01, 0.3, 0.42$ on the SLM before propagating the field to the target plane. SGD is thus oblivious to this model mismatch and we expect the results

to degrade with increasing σ as in conventional holographic displays. Our CITL approach uses the proposed camera-based forward model, which perfectly describes the physical optical image formation, including SLM phase noise, as captured by the camera, but it uses the same approximated gradients as SGD for the error backpropagation pass. We expect CITL to be better than SGD, because it uses a more accurate forward model, which is what we have observed in all of our experimental results so far.

Experiment 1 (SLED). Our first experiment, shown on the left of Figure S6, aims to answer question (1): what is the optimal bandwidth of an SLED? We simulate varying bandwidths of a collimated SLED source, measured as full width at half maximum (FWHM) of the SLED's spectral emission profile for 3 different amounts of model mismatch σ. As expected, when the model mismatch is very low ($\sigma = 0.01$), i.e., the difference between simulated and physical wave propagation is small, both SGD and CITL perform roughly equal. In this case, there is probably no need to use a CITL approach, as the physical optics can be perfectly modeled. For an increasing bandwidth, the performance of both SGD and CITL drops, as it becomes increasingly difficult to correct the blur introduced by the increasing spectral bandwidth of the source; remember, this is an ill-posed inverse problem. For larger amounts of model mismatch ($\sigma = 0.3, 0.42$), however, CITL outperforms SGD by a large margin – about 10 dB of PSNR. The model mismatch results in speckle when the SLED has a very narrow bandwidth, which is mostly but not fully corrected by the CITL optimization approach. Although the forward model for CITL matches the physical optics, the approximated gradients prevent CITL from achieving optimal results for very narrow bandwidths – there will always be some speckle. This is exactly the effect we observe in our experimental results with the laser. As the bandwidth of the SLED increases, the system becomes more tolerant to a model mismatch and achieves better results. Nevertheless, as the bandwidth increases further, it becomes increasingly difficult to solve the ill-posed inverse problem. We note that a bandwidth of approx. 3–5 nm seems to be optimal for the experimental conditions simulated here. This is lower than the bandwidth of 15 nm we determined to be optimal in Table 1 of the main paper. This difference is due to the way we simulate the model mismatch in this experiment and that observed in our physical display. Moreover, the fact that the optimal bandwidth changes for both conditions indicates that best spectral bandwidth of an SLED really does depend on the specific display and its particular optical characteristics. Also see Figure S7 for qualitative results of this experiment for $\sigma = 0.42$.

Experiment 2 (LED with single wavelength). Our second experiment, shown in the center of Figure S6, aims to answer question (2): what is the optimal pinhole size for an LED with an extremely narrow bandwidth? This experiment is a bit contrived, because LEDs typically do not have such narrow bandwidths, but the experiment is still educational. Unsurprisingly, we observe the same trends as for experiment 1. Varying the spatial coherence of this source leads to a tradeoff between speckle and blur whenever there is a model mismatch between simulated wave propagation and physical optics. The optimal pinhole size is somewhere between 25–35 μm for this experiment, but the optimal diameter depends on the specific amount of model mismatch. This experiment thus confirms that the insights from our first experiment, testing temporal coherence, carry over to spatial coherence. Also see Figure S8 (top row) for qualitative results of this experiment.

Experiment 3 (LED with 5 nm bandwidth). Our third experiment, shown on the right of Figure S6, aims to answer question (3): what is the optimal pinhole size for an LED with a moderate bandwidth? This is a more realistic scenario than experiment 2, because we now picked a bandwidth of 5 nm for the LED, as determined for the SLED in these simulated experimental conditions in experiment 1, but we also vary the pinhole size, as in experiment 2. We observe that smaller pinhole sizes are always better than larger sizes using the CITL approach. Also see Figure S8 (bottom row) for qualitative results of this experiment.

These three experiments indeed validate the primary conclusions of our paper: SLEDs, which are spatially coherent (i.e., collimated) and thus do not require a pinhole, always achieve a better quality than LEDs, which are spatially and temporally incoherent. We also confirm that the SLED is the best choice, even over a coherent laser, whenever there is some amount of mismatch between the simulated wave propagation model and the physical optics. If the model perfectly matches the physical optics, however, then a laser is the best choice. However, we have not been able to perfectly calibrate an analytic wave propagation model in our experimental settings, nor have we seen that in

the literature. Therefore, SLEDs seem like the ideal light sources for practical holographic displays.

Please note that whenever a pinhole is used, as simulated here for the LEDs, the light efficiency of the optical system suffers, which is a severe problem in practice, especially for optical see-through augmented reality displays. We did not take this loss of light efficiency into account for our quality metrics. Doing so would emphasize our conclusion that SLEDs are ideal light sources for holographic displays even more. Also, we simulated the mismatch between simulated model and physical optics as phase noise on the SLM whereas this would result from a variety of different factors, including phase nonlinearity, optical aberrations, and undiffracted light, in practice. Therefore, the specific numbers of optimal bandwidth for the SLEDs and pinhole size for LEDs derived here should not be taken too literally, although the trends certainly apply in different experimental conditions.

Verification of CITL improvement In Figure S9, we present the results obtained with the same setting using our full partially coherent modeling without and with CITL calibration. These results are obtained using LED on single channel (green) and visualized as grayscale images.

Resolution assessment To intuitively show the perceived resolution of partially coherent holography, we present the results of USAF-1951 resolution chart for the green channel, as shown in Figure S10. We observe resolution and contrast loss (see the cropped and zoomed patches) in the result of LED. However, for block regions with relatively uniform target intensities, the two partially coherent holography results show much less speckle artifact, with the SLED preserving the sharpness comparable as that of laser. The SLM phase patterns corresponding to these results are shown in Figure S20.

Additional comparison results of laser-, LED-, and SLED-based CITL holography We present additional comparison results of laser-, LED-, and SLED-based CITL holography, as shown in Figure S12, S14, and S16. We recommend readers to zoom in for distinguishing the difference in perceptual resolution and speckle level. The SLM phase patterns corresponding to all of these results are shown in Figures S13, S15, and S17, respectively.

Additional comparison results of LED- and SLED-based CITL holography We present additional comparison results of LED- and SLED-based CITL holography, as shown in Figure S18. We recommend readers to zoom in for distinguishing the difference in perceptual resolution and speckle level. In addition, we show that out SLED-based CITL holography is able to deliver great image quality on realistic 2D scenes, as shown in Figure S19. Their corresponding phases to display on SLM are show in Figure S20.

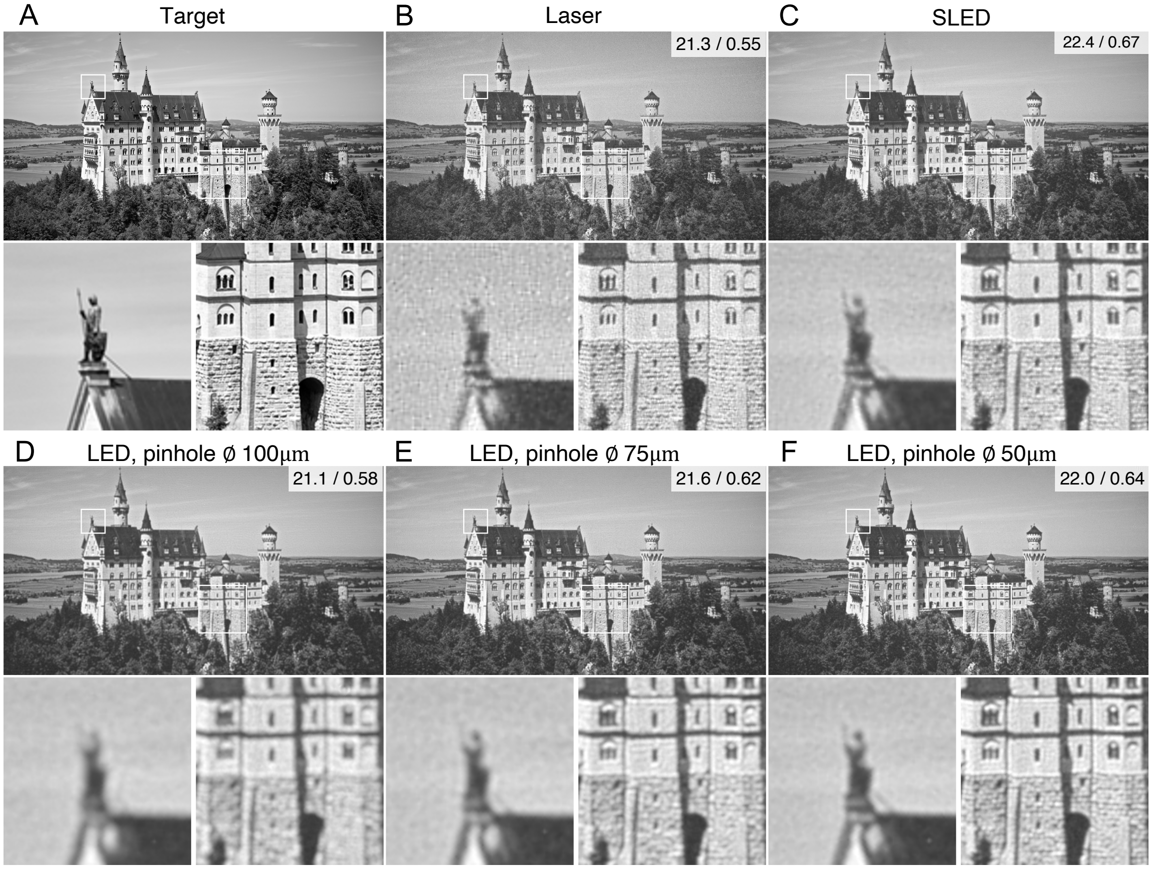

Fig. S4. Experimental comparisons of different light sources with varying pinhole sizes. The used light sources include the laser (B), SLED (C), and LED (D–F). All these results are captured with CITL calibration on one single channel (green). The target is shown (A). Quantitative results indicate the PSNR and SSIM, respectively. Image Credit: Eirikur Agustsson, Radu Timofte, and Rachel Davis.

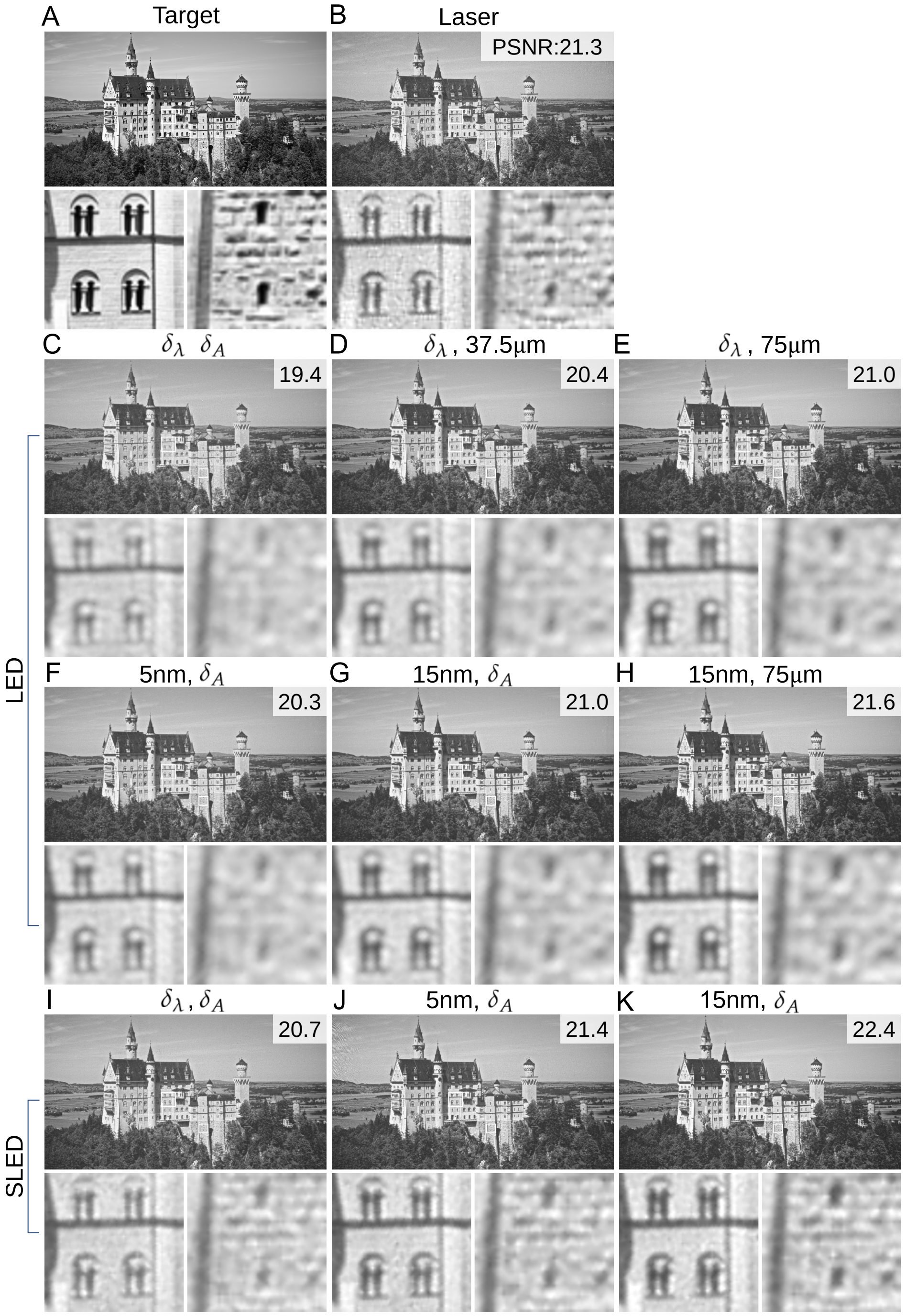

Fig. S5. Experimental ablation study on sampling diversity of wavelengths and angles. For each sub-figure, we show peak signal-to-noise ratio (PSNR) in addition to the captured images. Here, a Dirac delta δ_A represents the sampling of a single angle, which is equivalent to the assumption that the incident beam is emitted from one single point and well collimated. *37.5µm* and *75µm* represent the sampling over a finite-sized spot of 37.5 μm and 75 μm, respectively. The latter matches the physical size of the pinhole placed in the prototype display. A Dirac delta δ_λ represents the sampling of one single wavelength, *5nm* and *15nm* represent the sampling over the spectra of 5 nm and 15 nm, respectively. Again, all these results are obtained with CITL calibration on one single channel (green). Image Credit: Eirikur Agustsson, Radu Timofte, and Rachel Davis.

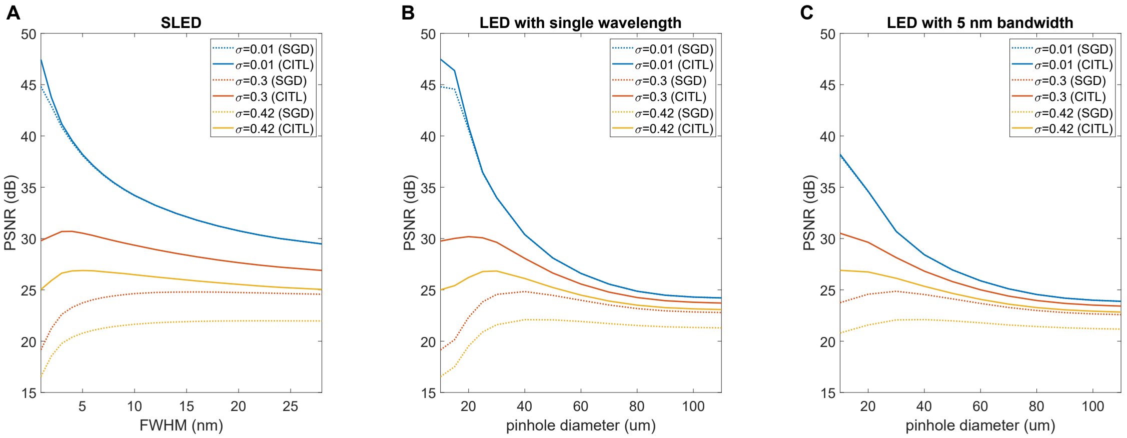

Fig. S6. Analyzing SLED bandwidth and LED pinhole size. (A) An SLED is collimated and does not require a pinhole. The CITL optimization approach is strictly better than an SGD approach for any amount of mismatch between the simulated wave propagation model and the physical optics. The optimal bandwidth of the SLED is between 3–5 nm. (B) The spatial coherence of an LED with a very narrow bandwidth can be controlled by a pinhole. Similar to the varying temporal coherence of the SLED, this results in a tradeoff between speckle and blur whenever there is a model mismatch. (C) For an LED with a bandwidth of 5 nm, larger pinholes always reduce the image quality. Note that using a pinhole with an LED always reduces the light efficiency of the system compared to the SLED; this degradation is not taken into account for the PSNR reported with these experiments.

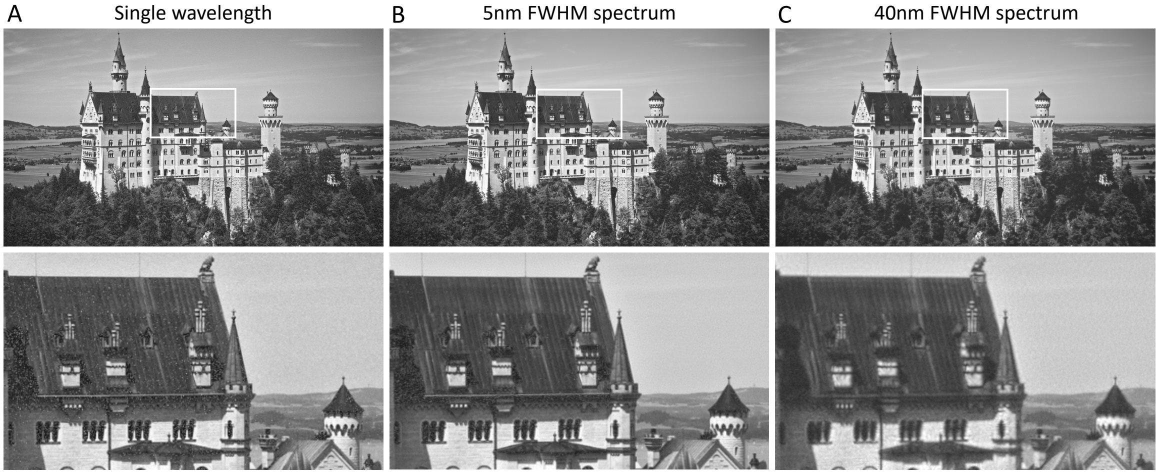

Fig. S7. Analyzing SLED bandwidth in simulation. When the bandwidth is just a single wavelength, as is the case for a coherent laser, we observe speckle when the simulated wave propagation model does not exactly match the physical optics (A). An SLED with a small bandwidth can optimize the image quality (B). As the bandwidth increases, the blur introduced by the optical image formation becomes too severe to be compensated by the algorithm (C). All of these results are optimized with the proposed CITL approach. Image Credit: Eirikur Agustsson, Radu Timofte, and Rachel Davis.

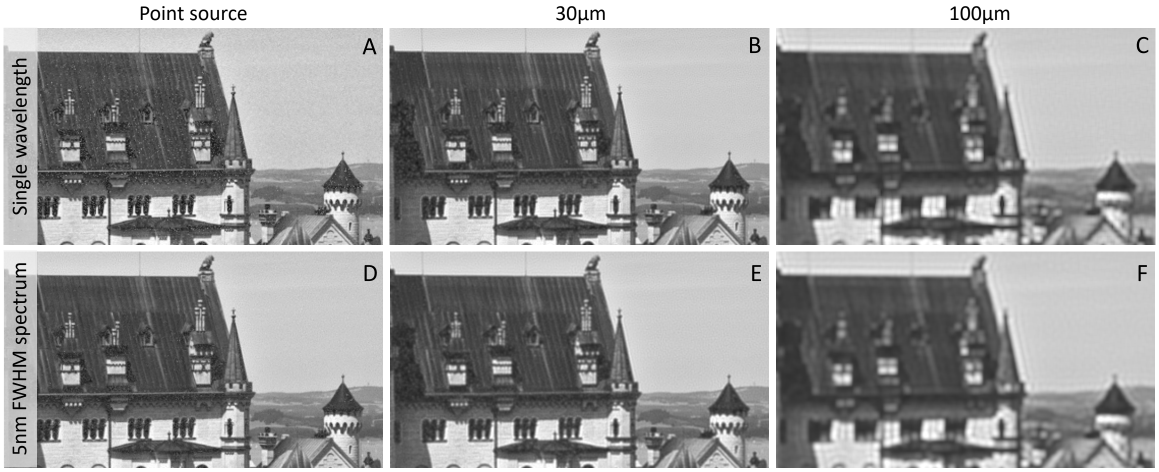

Fig. S8. Analyzing LED bandwidth and pinhole size in simulation. (A–C) For an LED emitting just a single wavelength over a finite area, a pinhole can control its spatial coherence. A smaller pinhole diameter makes the source more coherent, but results in speckle, whereas a larger pinhole naturally blurs out the speckle but at the cost of degraded image sharpness. (D–F) For an LED with a small bandwidth of 5 nm, a smaller pinhole always results in better image quality. All of these results are optimized with the proposed CITL approach. Image Credit: Eirikur Agustsson, Radu Timofte, and Rachel Davis.

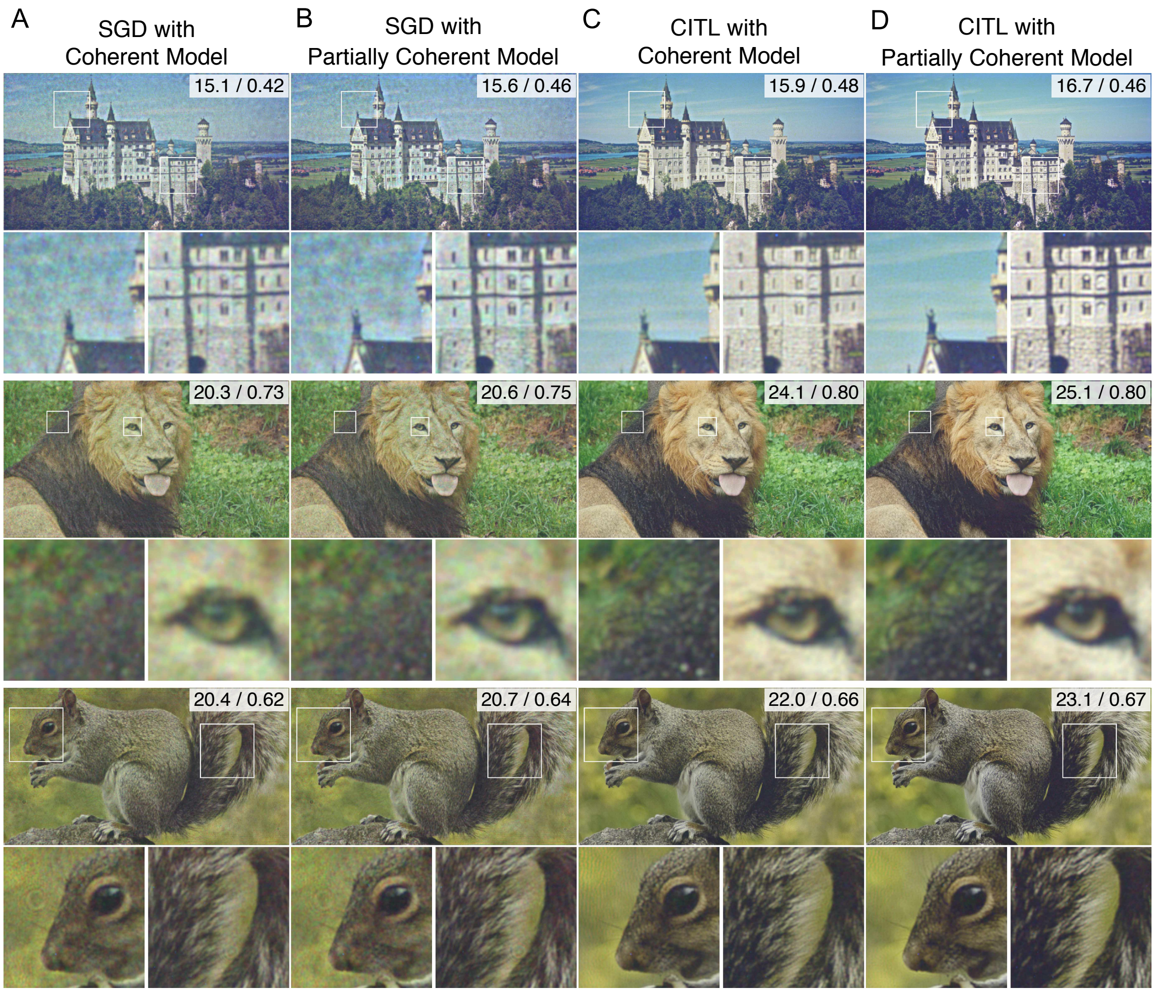

Fig. S9. Comparison of results of partially coherent holography using LED. From left to right (A–D) are SGD + naive ASM, SGD + partially coherent model, CITL + naive ASM, and CITL + partially coherent model, respectively. Quantitative results indicate the PSNR and SSIM. Images Credits: Eirikur Agustsson, Radu Timofte, Rachel Davis, Pikist, and Alastair Newton.

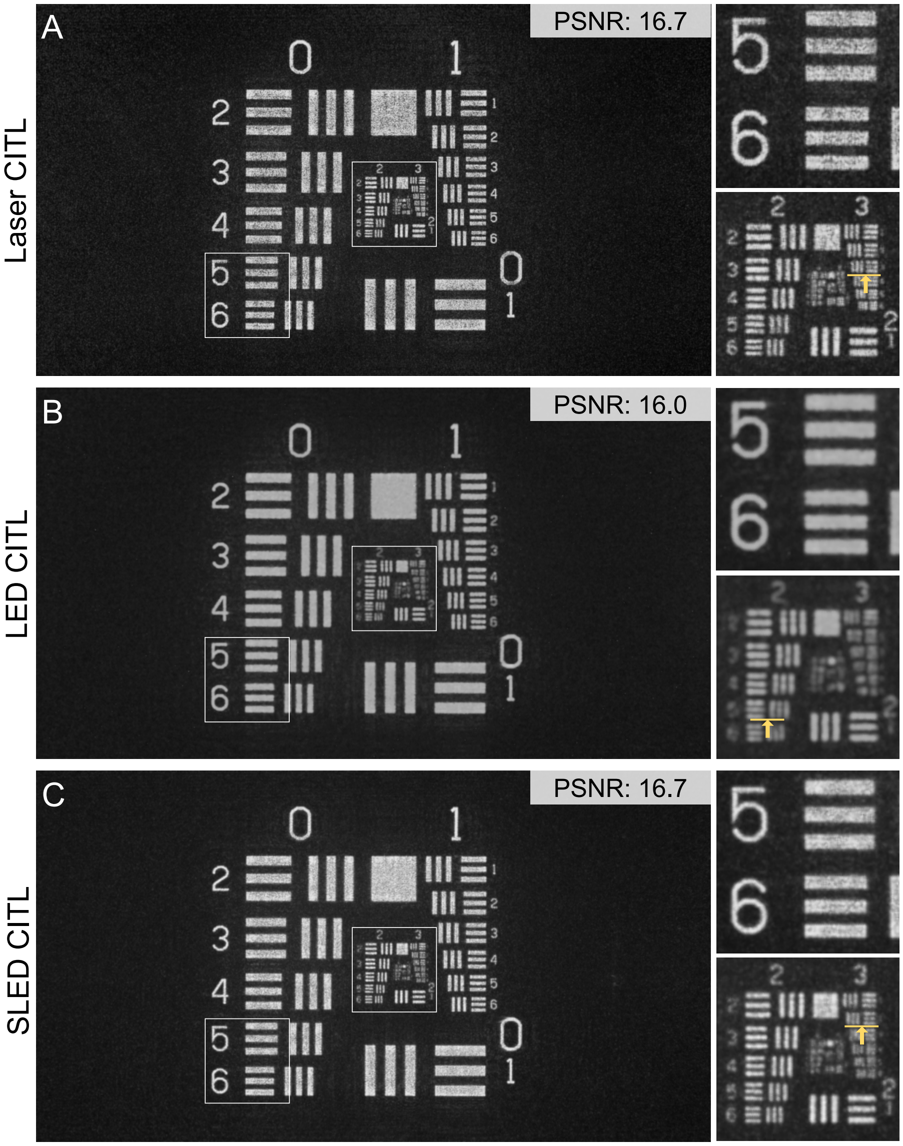

Fig. S10. Experimental results of USAF-1951 resolution chart reconstructed with different light sources. The light sources include the laser (A), LED (B), and SLED (C). Quantitative results indicate the PSNR. The equivalent perceptual resolution of three examples are approximately 20 μm, 32 μm, and 23 μm, respectively, as indicated by the arrows.

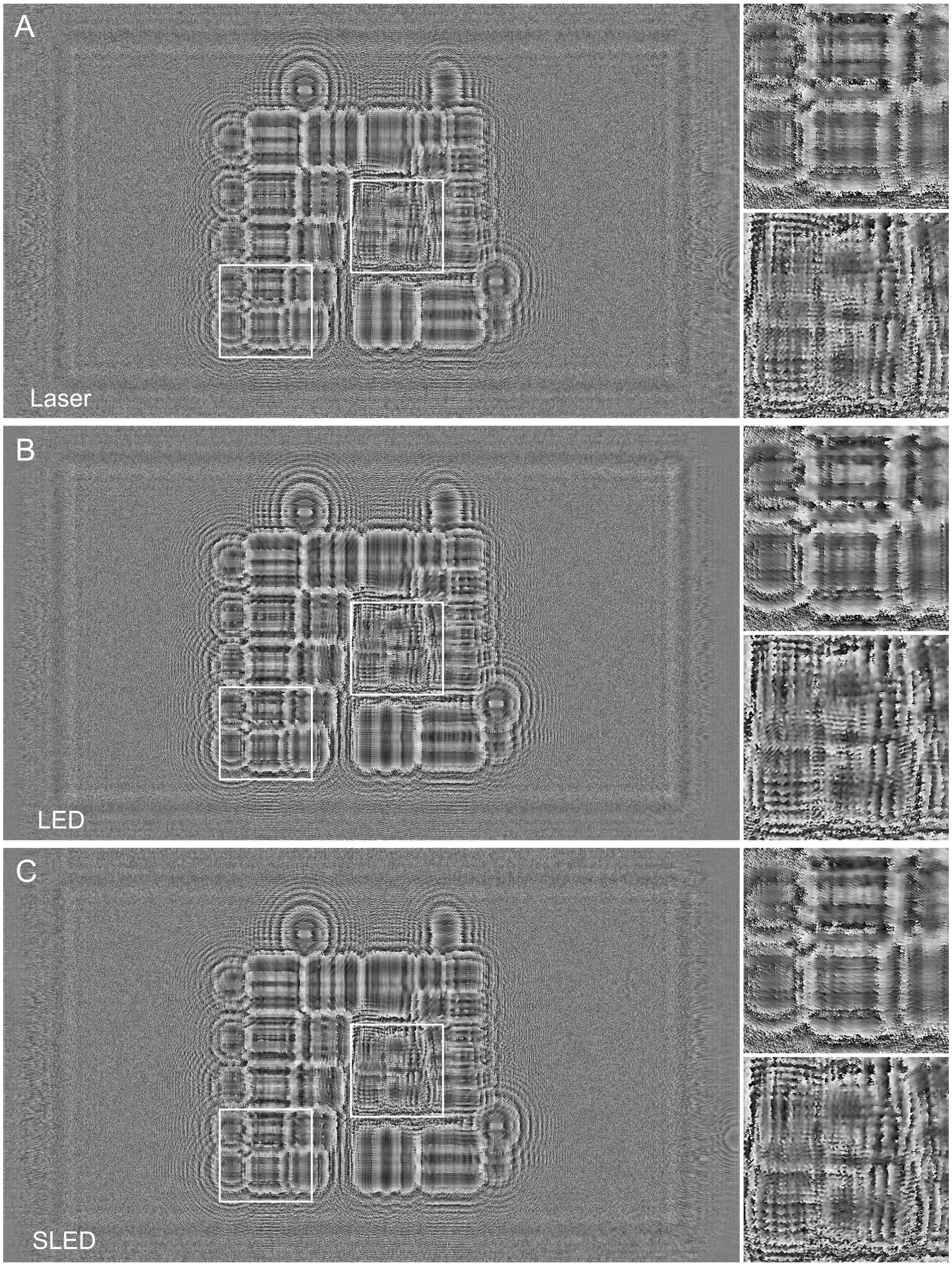

Fig. S11. SLM phase patterns of the green color channel for results shown in Figure S10.

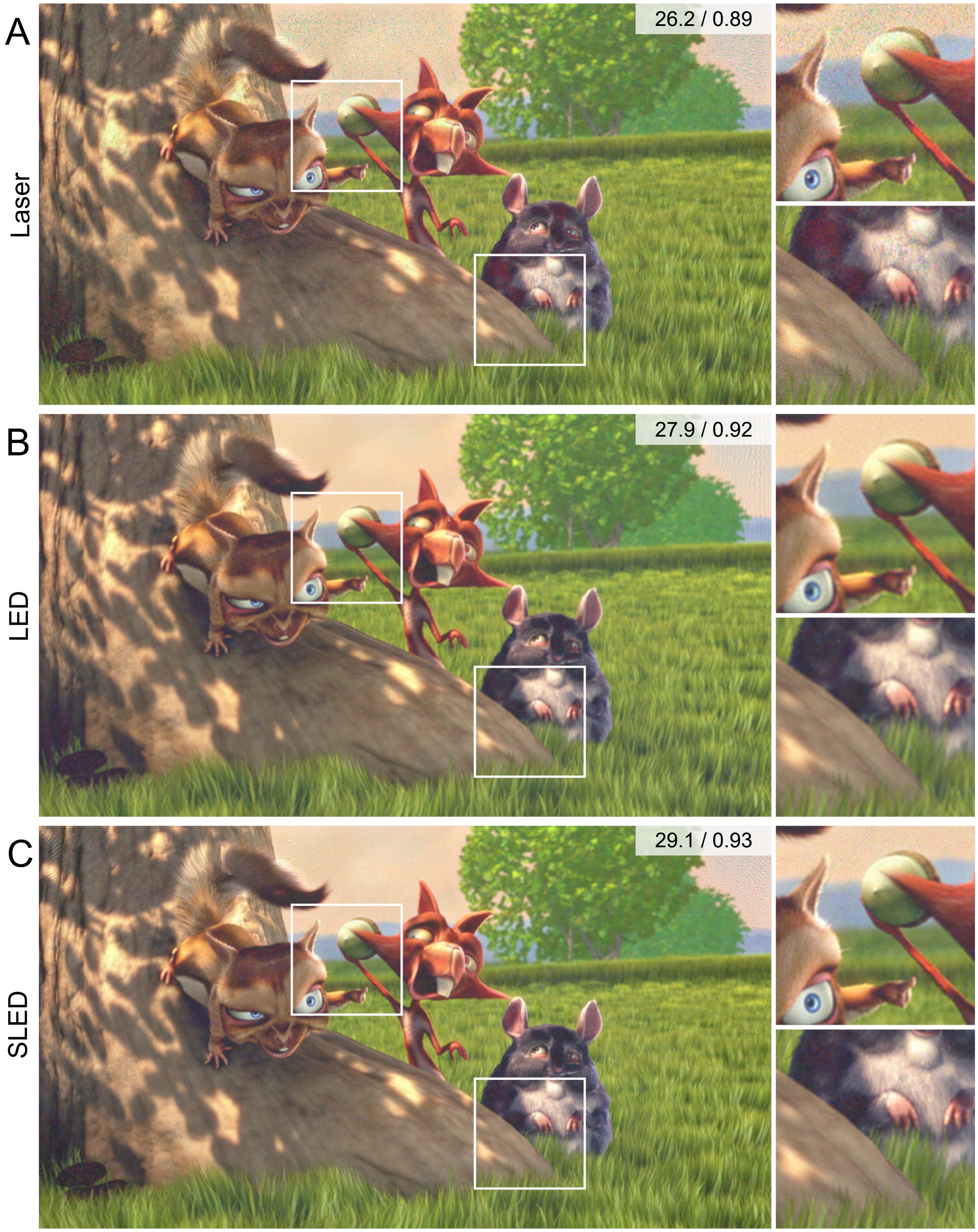

Fig. S12. Experimental results of images reconstructed with different light sources. The light sources include the laser (A), LED (B), and SLED (C), all with the CITL calibration. Quantitative results indicate the PSNR and SSIM. Image Credit: Big Buck Bunny, Blender Institute.

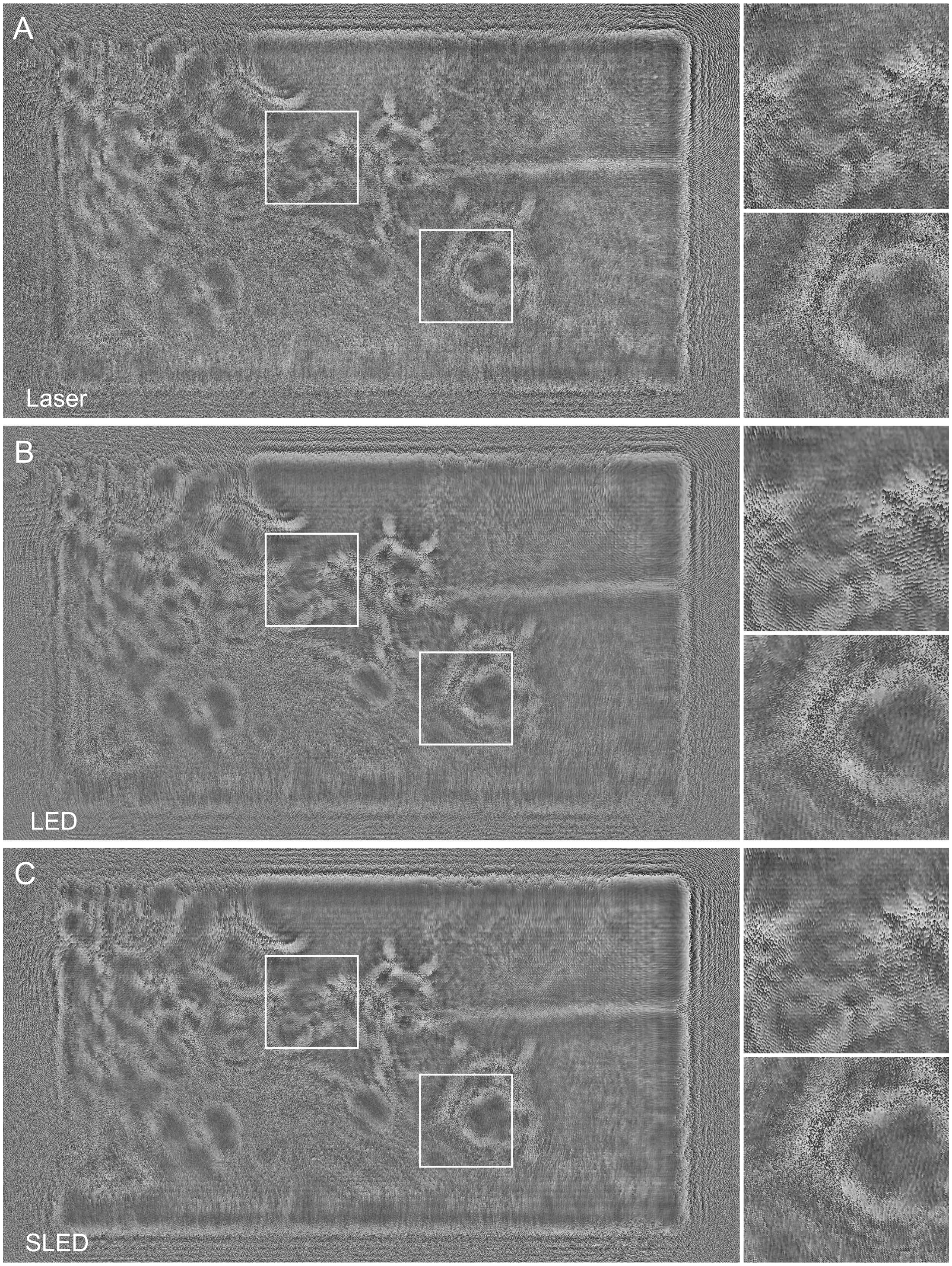

Fig. S13. SLM phase patterns of green color channel of results shown in Figure S12.

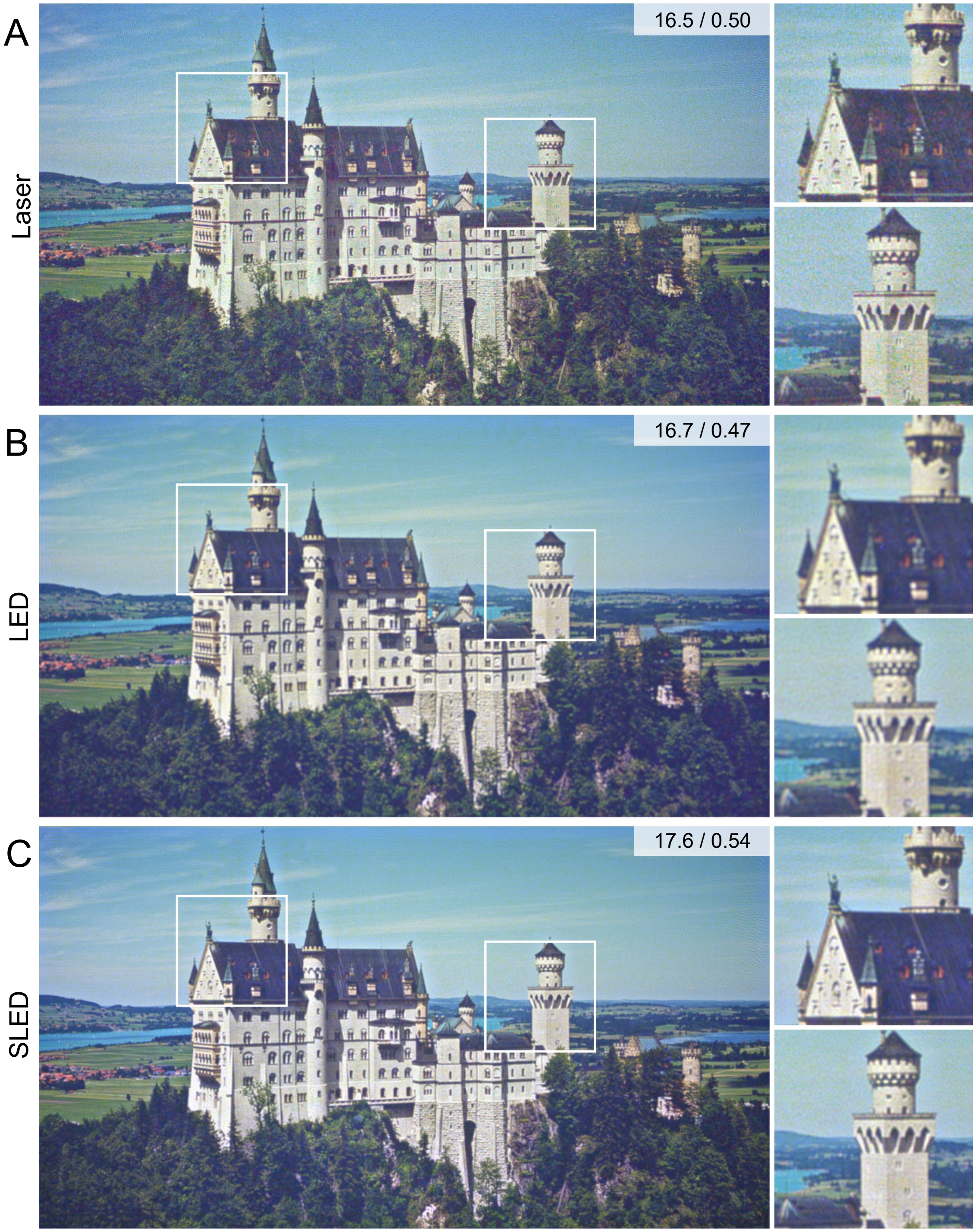

Fig. S14. Experimental results of images reconstructed with different light sources. The light sources include the laser (A), LED (B), and SLED (C), all with the CITL calibration. Quantitative results indicate the PSNR and SSIM. Image Credit: Eirikur Agustsson, Radu Timofte, and Rachel Davis.

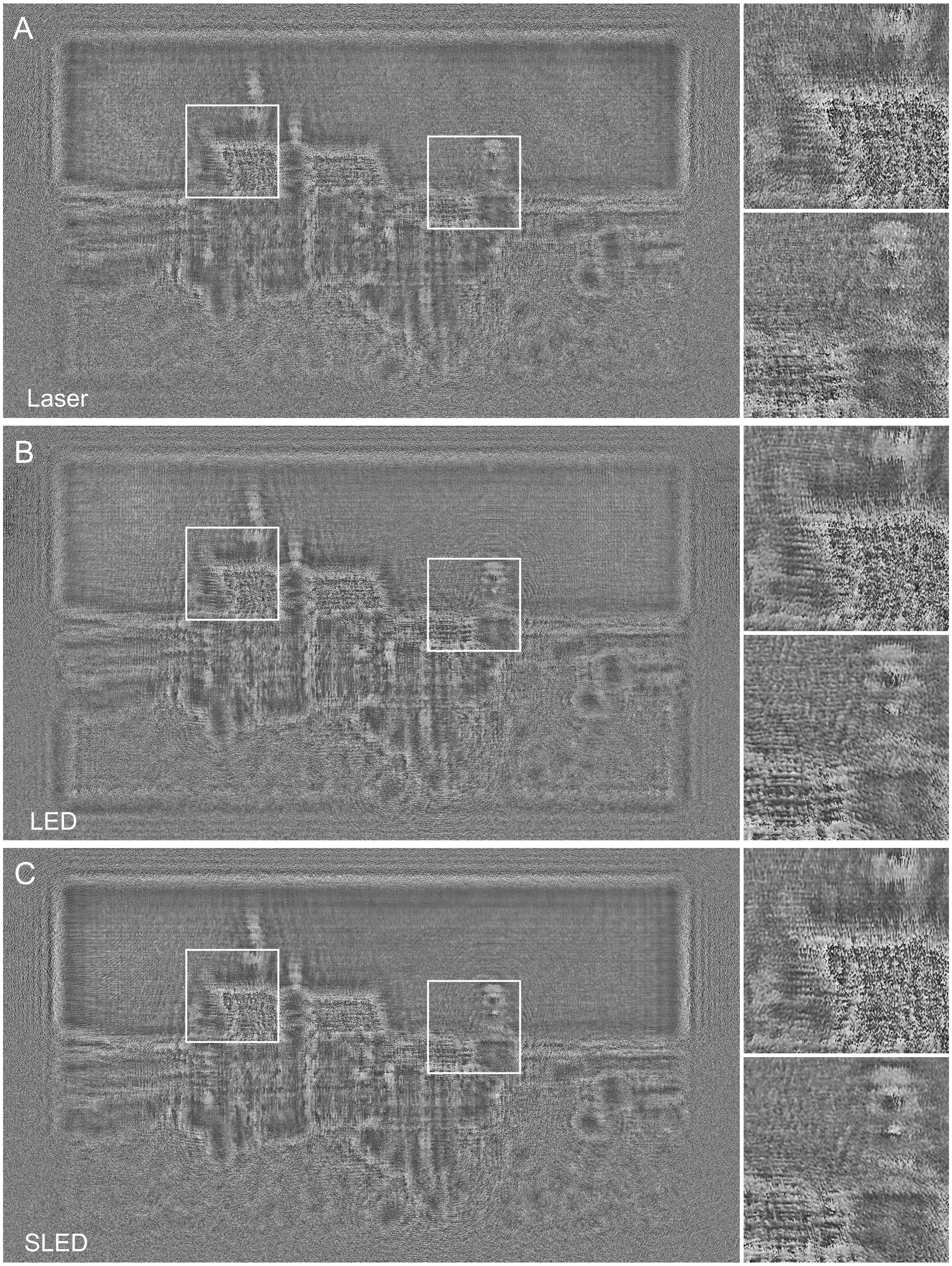

Fig. S15. SLM phase patterns of green color channel of results shown in Figure S14.

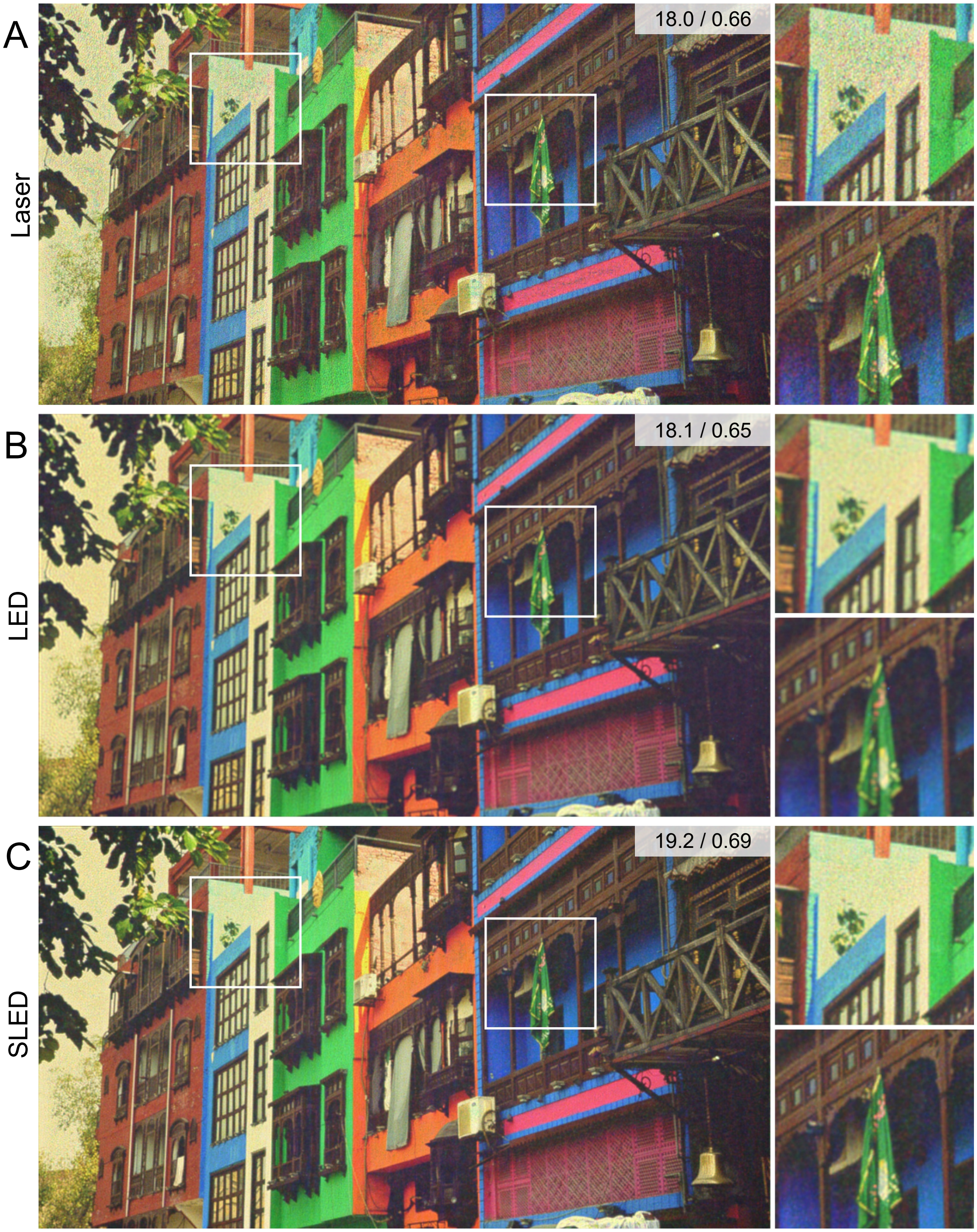

Fig. S16. Experimental results of images reconstructed with different light sources. The light sources include the laser (A), LED (B), and SLED (C), all with the CITL calibration. Quantitative results indicate the PSNR and SSIM. Image Credit: Eirikur Agustsson, Radu Timofte, and Sabah Sarwar.

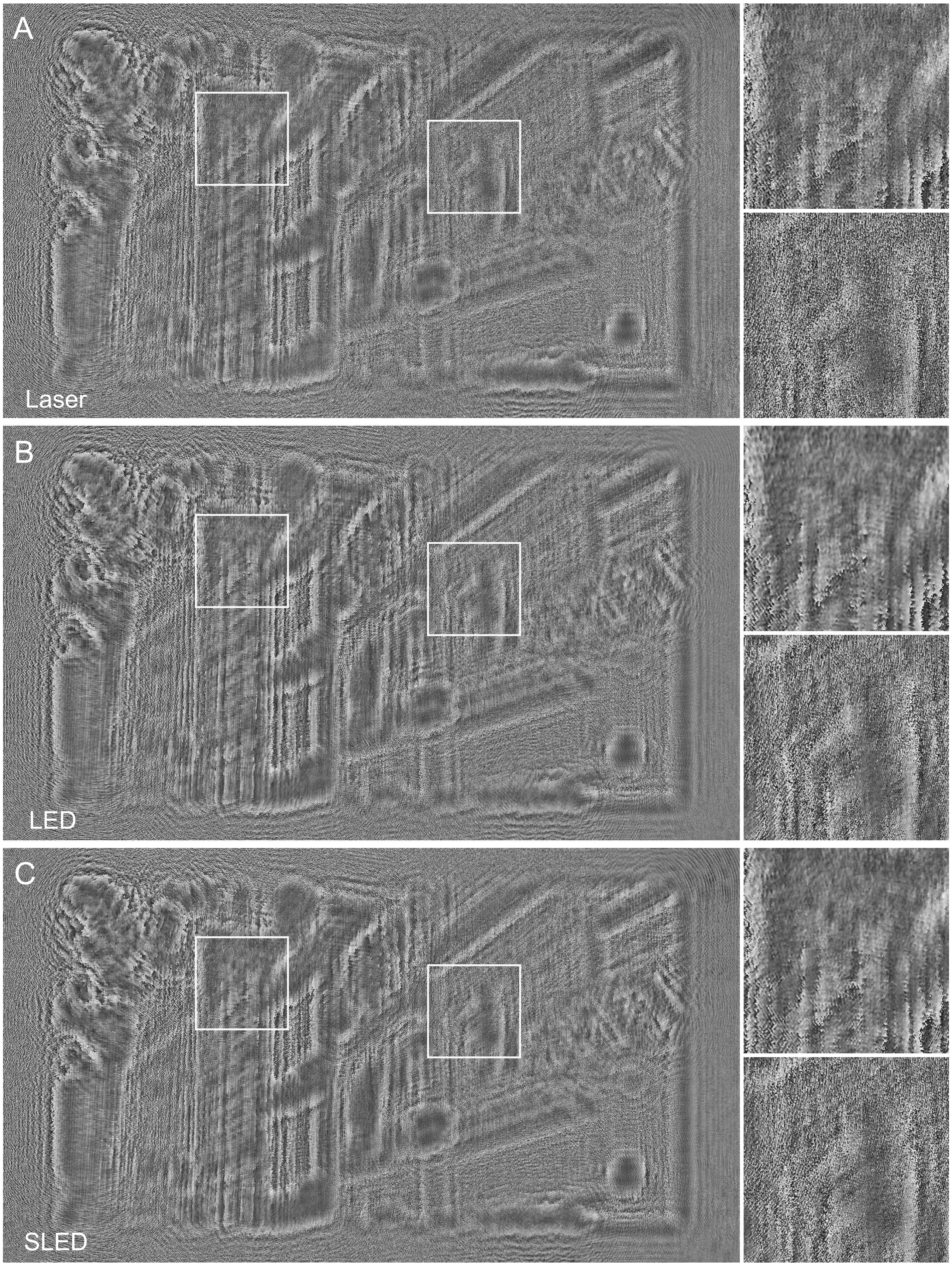

Fig. S17. SLM phase patterns of green color channel of results shown in Figure S16.

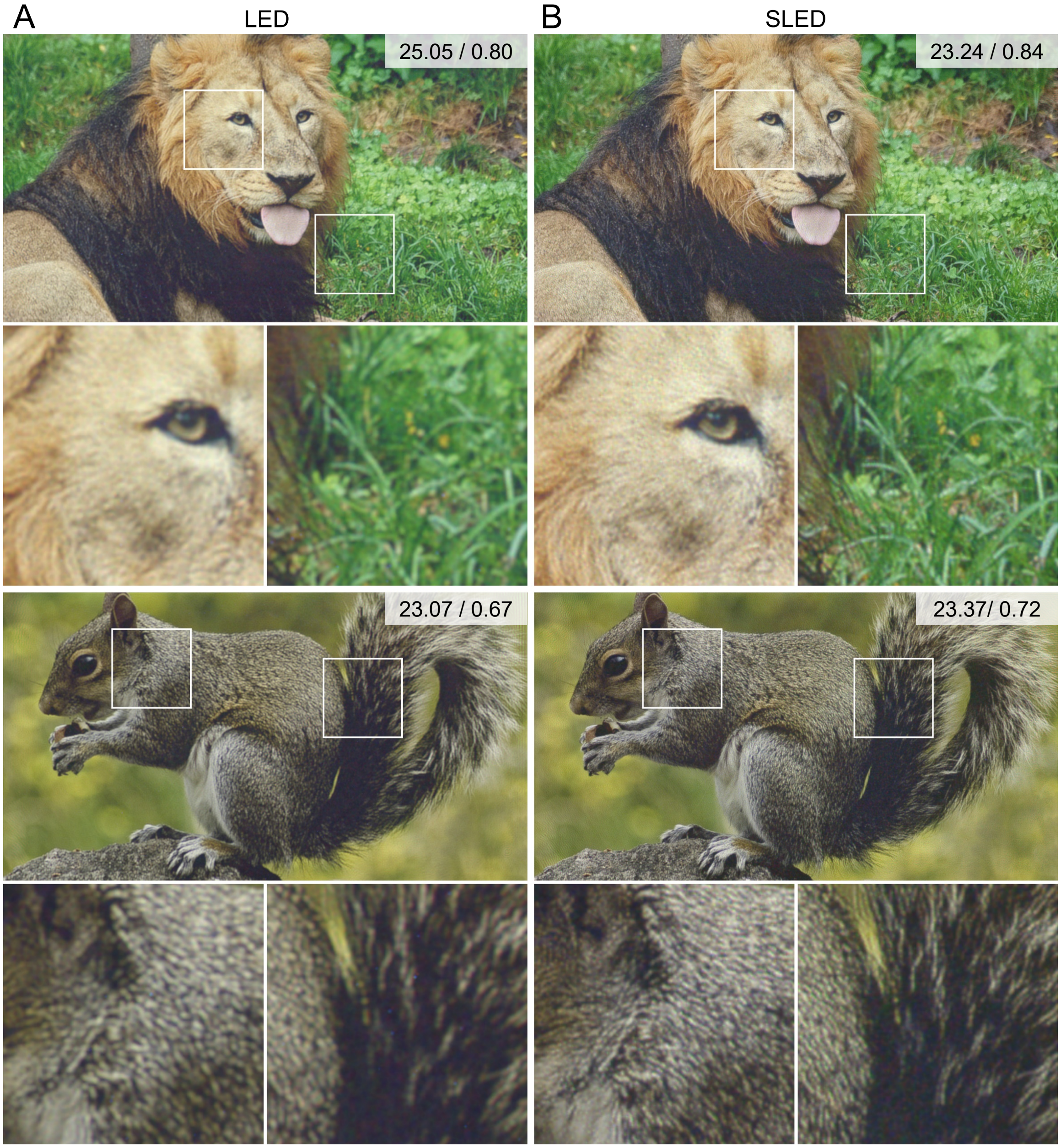

Fig. S18. Experimental results of images reconstructed with different partially coherent light sources. The light sources include the LED (A) and SLED (B), both with the CITL calibration. Quantitative results indicate the PSNR and SSIM. Images Credits: Eirikur Agustsson, Radu Timofte, Pikist, and Alastair Newton.

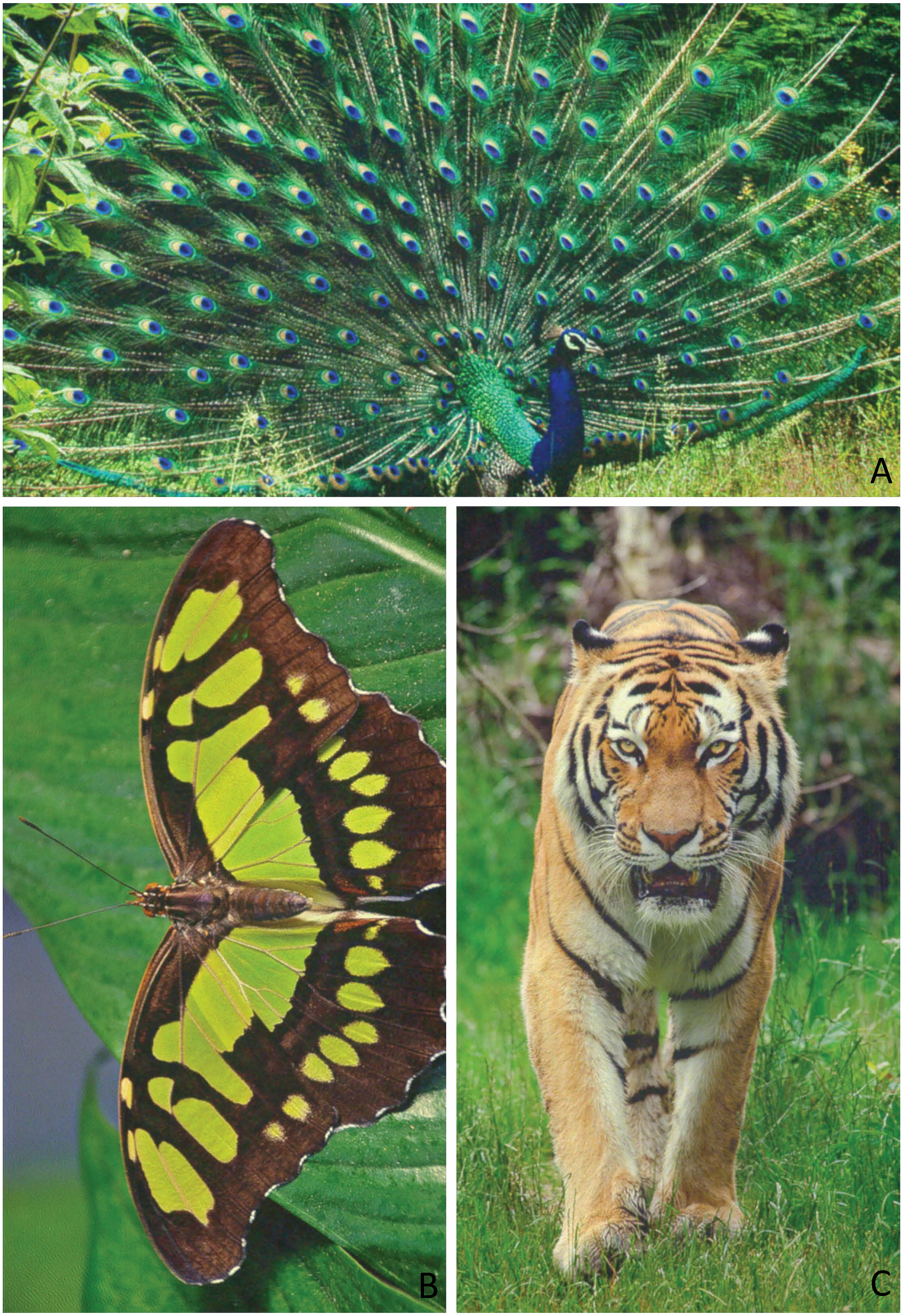

Fig. S19. Experimentally captured results reconstructed with SLED and the CITL calibration. Images Credits: Eirikur Agustsson, Radu Timofte, N. A. Nasee, Pixabay, and PxHere.

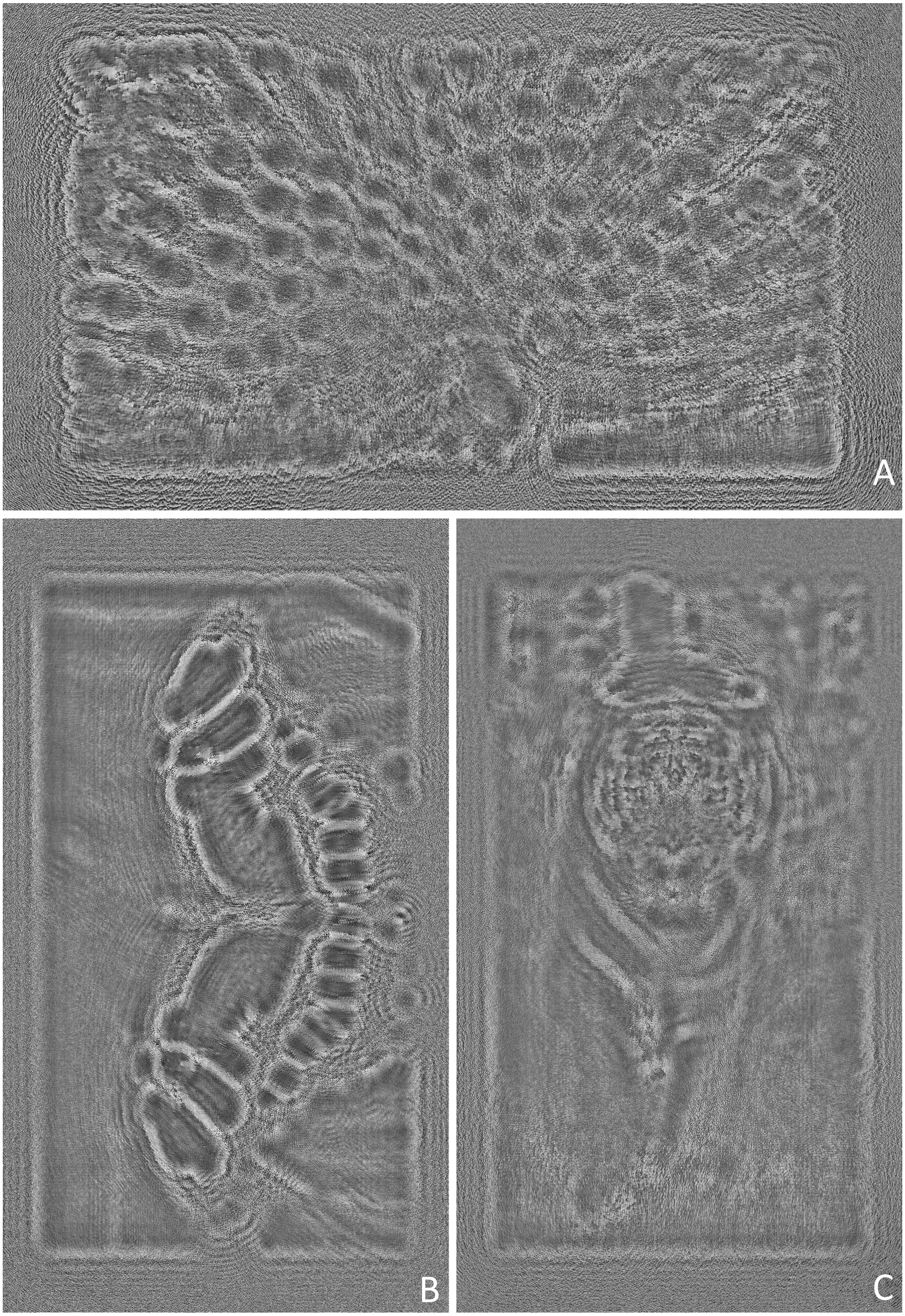

Fig. S20. SLM phase patters of green color channel for results shown in Figure S19.

REFERENCES AND NOTES

1. F. L. Kooi, A. Toet, Visual comfort of binocular and 3D displays. *Displays* **25**, 99–108 (2004).

2. M. Lambooij, M. Fortuin, I. Heynderickx, W. IJsselsteijn, Visual discomfort and visual fatigue of stereoscopic displays: A review. *J. Imaging Sci. Technol.* **53**, 30201-1–30201-14 (2009).

3. C. Chang, K. Bang, G. Wetzstein, B. Lee, L. Gao, Toward the next-generation vr/ar optics: A review of holographic near-eye displays from a human-centric perspective. *Optica* **7**, 1563–1578 (2020).

4. R. Jones, C. Wykes, J. Wykes, in *Holographic and Speckle Interferometry* (Cambridge Univ. Press, 1989), vol. 6.

5. J. C. Dainty, in *Laser Speckle and Related Phenomena* (Springer Science & Business Media, 2013), vol. 9.

6. K. V. Chellappan, E. Erden, H. Urey, Laser-based displays: A review. *Appl. Optics* **49**, F79–F98 (2010).

7. D.-Y. Park, J.-H. Park, Hologram conversion for speckle free reconstruction using light field extraction and deep learning. *Opt. Express* **28**, 5393–5409 (2020).

8. V. Bianco, P. Memmolo, M. Leo, S. Montresor, C. Distante, M. Paturzo, P. Picart, B. Javidi, P. Ferraro Strategies for reducing speckle noise in digital holography. *Light Sci. Appl.* **7**, 48 (2018).

9. J. W. Goodman, Speckle: Friend or foe? *AIP Conf. Proc.* **1537**, 5–7 (2013).

10. J.-W. Pan, C.-H. Shih, Speckle reduction and maintaining contrast in a laser pico-projector using a vibrating symmetric diffuser. *Opt. Express* **22**, 6464–6477 (2014).

11. M. N. Akram, Z. Tong, G. Ouyang, X. Chen, V. Kartashov, Laser speckle reduction due to spatial and angular diversity introduced by fast scanning micromirror. *Appl. Optics* **49**, 3297–3304 (2010).

12. T.-K.-T. Tran, X. Chen, Ø. Svensen, M. N. Akram, Speckle reduction in laser projection using a dynamic deformable mirror. *Opt. Express* **22**, 11152–11166 (2014).

13. D. Lee, C. Jang, K. Bang, S. Moon, G. Li, B. Lee Speckle reduction for holographic display using optical path difference and random phase generator. *IEEE Trans. Industr. Inform.* **15**, 6170–6178 (2019).

14. Y. Deng, D. Chu, Coherence properties of different light sources and their effect on the image sharpness and speckle of holographic displays. *Sci. Rep.* **7**, 5893 (2017).

15. N. Primerov, J. Dahdah, S. Gloor, N. Matuschek, Tim von Niederhäusern, A. Castiglia, M. Malinverni, C. Mounir, M. Rossetti, M. Duelk, C. Vélez, P-203: Late-news poster: Integrated full-color rgb superluminescent led module for micro-displays. *SID Symp. Dig. Tech. Pap.* **50**, 1731–1734 (2019).

16. E. Moon, M. Kim, J. Roh, H. Kim, J. Hahn, Holographic head-mounted display with rgb light emitting diode light source. *Opt. Express* **22**, 6526–6534 (2014).

17. F. Yaraş, H. Kang, L. Onural, Real-time phase-only color holographic video display system using led illumination. *Appl. Optics* **48**, H48–H53 (2009).

18. M. Askari, J.-H. Park, Pre-compensation of an image blur in holographic projection display using light emitting diode light source. *Opt. Express* **28**, 146–159 (2020).

19. S. Lee, D. Kim, S.-W. Nam, B. Lee, J. Cho, B. Lee, Light source optimization for partially coherent holographic displays with consideration of speckle contrast, resolution, and depth of field. *Sci. Rep.* **10**, 18832 (2020).

20. A. Maimone, A. Georgiou, J. S. Kollin, Holographic near-eye displays for virtual and augmented reality. *ACM Trans. Graph.* **36**, 85 (2017).

21. L. Shi, F.-C. Huang, W. Lopes, W. Matusik, D. Luebke, Near-eye light field holographic rendering with spherical waves for wide field of view interactive 3D computer graphics. *ACM Trans. Graph.* **36**, 1–17 (2017).

22. N. Padmanaban, Y. Peng, G. Wetzstein, Holographic near-eye displays based on overlap-add stereograms. *ACM Trans. Graph.* **38**, 1–13 (2019).

23. Y. Peng, S. Choi, N. Padmanaban, G. Wetzstein, Neural holography with camera-in-the-loop training. *ACM Trans. Graph.* **39**, 1–14 (2020).

24. L. Shi, B. Li, C. Kim, P. Kellnhofer, W. Matusik, Towards real-time photorealistic 3D holography with deep neural networks. *Nature* **591**, 234–239 (2021).

25. J. W. Goodman, *Introduction to Fourier Optics* (Roberts and Company Publishers, 2005).

26. K. Matsushima, T. Shimobaba, Band-limited angular spectrum method for numerical simulation of free-space propagation in far and near fields. *Opt. Express* **17**, 19662–19673 (2009).

27. S. Choi, J. Kim, Y. Peng, G. Wetzstein, Optimizing image quality for holographic near-eye displays with Michelson holography *Optica* **8**, 143–146 (2021).

28. W.-J. Joo, J. Kyoung, M. Esfandyarpour, S. H. Lee, H. Koo, S. Song, Y. N. Kwon, S. H. Song, J. C. Bae, A. Jo, M. J. Kwon, S. H. Han, S. H. Kim, S. Hwang, M. L. Brongersma Metasurface-driven oled displays beyond 10,000 pixels per inch. *Science* **370**, 459–463 (2020).

29. A. Paszke, S. Gross, F. Massa, A. Lerer, J. Bradbury, G. Chanan, T. Killeen, Z. Lin, N. Gimelshein, L. Antiga, A. Desmaison, A. Kopf, E. Yang, Z. De Vito, M. Raison, A. Tejani, S. Chilamkurthy, B. Steiner, L. Fang, J. Bai, S. Chintala, Pytorch: An imperative style, high-performance deep learning library. *Adv. Neural Inf. Process. Syst.* **32**, 8026–8037 (2019).

30. Y. Shechtman, Y. C. Eldar, O. Cohen, H. N. Chapman, J. Miao, M. Segev, Phase retrieval with application to optical imaging: A contemporary overview. *IEEE Signal Process. Mag.* **32**, 87–109 (2015).

31. A. W. Lohmann, S. Sinzinger, *Optical Information Processing* (1978).
